# Association between early-onset colorectal cancer outcomes and established prognostic factors: a nationwide population-based study

**DOI:** 10.1016/j.eclinm.2026.104270

**Published:** 2026-11-09

**Authors:** Lauri H. Borghuis, Sander R. van Hooff, Rik van Eekelen, Evelien Dekker, Louis Vermeulen, Tineke E. Buffart

**Affiliations:** a Laboratory of Experimental Oncology and Radiobiology, Cancer Center Amsterdam, Amsterdam UMC, Amsterdam, the Netherlands; b Oncode Institute, Amsterdam, the Netherlands; c Amsterdam Gastroenterology Endocrinology Metabolism, Amsterdam, the Netherlands; d Department of Epidemiology and Data Science, Amsterdam Public Health Research Institute, Amsterdam, the Netherlands; e Cancer Center Amsterdam, Amsterdam UMC, Amsterdam, the Netherlands; f Department of Gastroenterology & Hepatology, Amsterdam UMC, Amsterdam, the Netherlands; g Department of Medical Oncology, Amsterdam UMC, Amsterdam, the Netherlands

**Keywords:** Colorectal cancer, Early-onset, Age of onset, Relative survival, Molecular subtypes, Cancer heterogeneity

## Abstract

**Background:**

The incidence of early-onset colorectal cancer (eoCRC; <50 years) is rising. Previous studies suggested that eoCRC patients have poorer outcomes than older adults. Whether this reflects distinct tumor biology or differences in stage at diagnosis and treatment patterns remains unclear. We compared clinicopathological features, treatment, and survival of eoCRC with average-onset (ao) and older-onset (oo)CRC in a nationwide cohort.

**Methods:**

Clinicopathological data of all patients diagnosed with colorectal adenocarcinoma in the Netherlands from 1 January 2016 through 31 December 2022 were retrieved from the Netherlands Cancer Registry (NCR). Patients were categorized as eoCRC, aoCRC (50–69 years), and ooCRC (≥70 years). Multivariable regression and matched analyses were performed to account for established prognostic factors and predictors of treatment response.

**Findings:**

Among 80,551 patients with CRC, 3917 (5%) were eoCRC, 34,499 (43%) were aoCRC, and 42,135 (52%) were ooCRC. EoCRC patients more often presented with advanced disease (stage III or IV) and left-sided colon or rectal cancer. Five-year relative survival (RS) for eoCRC, aoCRC and ooCRC patients was 70%, 77%, and 69%, respectively (crude excess hazard ratio [EHR] for eoCRC vs. aoCRC 1·32 [95% CI 1·24–1·41]). In patients with mismatch repair proficient (pMMR) stage IV disease, eoCRC was associated with better RS than aoCRC (EHR 0·87 [95% CI 0·80–0·94]), concurrent with more intensive first-line systemic therapy. After matching for disease characteristics and treatment, RS was similar between eoCRC and aoCRC (EHR 1·02 [95% CI 0·91–1·14]), whereas ooCRC patients retained worse survival.

**Interpretation:**

The poorer crude survival observed in eoCRC is largely associated with more advanced disease at diagnosis, rather than evidence of intrinsically distinct tumor biology. After accounting for established prognostic factors and factors predicting treatment response, survival of eoCRC and aoCRC patients is similar, challenging the notion that eoCRC constitutes a separate biological disease entity.

**Funding:**

ZonMw, Dutch Research Council.

**Research in context**

**Evidence before this study**

The incidence of sporadic colorectal cancer (CRC) in patients younger than 50 years of age (early-onset CRC [eoCRC]) is increasing. Whether response to current systemic therapies differs between eoCRC and older patients, potentially reflecting underlying differences in tumor biology, remains unclear. We searched PubMed from 1 January 2016 to 31 August 2025 for cohort studies of real-world data on the comparison of treatment and survival of eoCRC and older CRC patients, using the terms ((early-onset colorectal cancer) AND (“older-onset colorectal cancer”) AND (“treatment”) AND (“survival”). Existing studies report conflicting results on whether eoCRC patients have worse survival compared to older patients (>50 years of age) and whether eoCRC patients should be treated differently. Studies directly comparing eoCRC patients with two distinct older age groups, matched for clinical characteristics, are lacking.

**Added value of this study**

To our knowledge, this is the first large nationwide, population-based study using real-world data to compare treatment and survival of eoCRC patients with average-onset (aoCRC; 50–69 years) and older-onset (ooCRC; ≥70 years) patients. By applying a comprehensive matching strategy across age groups for established prognostic and predictive factors, we were able to provide a more accurate comparison of outcomes than previous reports. In line with previous reports, we observed that eoCRC patients have worse survival than aoCRC patients when all tumor stages are combined, however, eoCRC patients with stage IV disease have better survival. After matching for clinical characteristics and treatment, survival of eoCRC and aoCRC patients is comparable, while survival of ooCRC patients remains worse.

**Implications of all the available evidence**

Our nationwide study shows that the poorer survival of patients with eoCRC is largely attributable to more advanced disease at diagnosis, with survival similar to that of patients with aoCRC after adjustment for established prognostic and predictive clinical variables. These findings question the notion that eoCRC constitutes a distinct biological entity and suggest that efforts to improve outcomes in eoCRC should prioritize earlier detection and prevention.

## Introduction

The incidence of colorectal cancer (CRC), one of the leading causes of cancer-related death worldwide, has declined over the past decade following the implementation of screening programs.^1^, ^2^ In contrast, the incidence of early-onset CRC (eoCRC), defined as diagnosis <50 years, is rising.^3^, ^4^, ^5^ CRC is now the leading cause of cancer-related death in young men and the second leading cause in young women.^6^

A minority of eoCRC cases (16–25%) are attributable to hereditary cancer syndromes, including Lynch syndrome, familial adenomatous polyposis (FAP) or MUTYH associated polyposis.^4^ The majority of eoCRC cases are sporadic, and are likely influenced by environmental and behavioral factors, including diet and sedentary lifestyle. However, the precise causes and mechanisms driving the increase in eoCRC remain unclear.^7^

Existing studies report conflicting results on whether eoCRC patients have worse overall survival (OS) than older patients and whether they require different treatment strategies.^8^ EoCRC is more often diagnosed at an advanced stage, more frequently involves left-sided colon or rectum, and is more often poorly differentiated.^9^, ^10^ Furthermore, high-risk stage III early-onset colon cancer has been associated with higher recurrence rates despite adjuvant therapy.^11^ Several studies have reported differences in tumor biology between young and older adults with CRC, including differences in the frequency of driver mutations and tumor expression profiles.^12^, ^13^, ^14^ It remains unclear to what extent tumor location, stage, and molecular tumor characteristics explain survival differences between eoCRC and patients aged 50 years or older.

Young adults with CRC are generally treated similarly to patients aged 50 years or older, as evidence supporting age-specific treatment approaches is limited. Most pivotal trials guiding current treatment recommendations predominantly enrolled patients aged 50–70 years (average-onset CRC; aoCRC), leaving little prospective evidence specific to eoCRC.^15^, ^16^ An age threshold of 70 years or older is commonly used to define older-onset CRC (ooCRC), reflecting increasing frailty, comorbidity, and the impact of treatment on quality of life.^17^, ^18^ Consequently, older patients may be undertreated, whereas younger patients may receive more intensive treatment, with a potential risk of overtreatment.^19^, ^20^

The key unresolved question is whether tumor response to current systemic therapies truly differs between younger and older patients, potentially reflecting underlying differences in tumor biology. In this study, we leveraged data from the nationwide Netherlands Cancer Registry (NCR) to compare clinical characteristics, treatment patterns, and survival of patients with eoCRC, aoCRC, and ooCRC. Our aim was to determine whether eoCRC represents a distinct clinical entity with different outcomes after accounting for established prognostic factors and factors predicting treatment response.

## Methods

### Data collection

Data on all patients 18 years or older who were diagnosed with adenocarcinoma of the colon or rectum, according to the International Classification of Disease for Oncology codes C18 (colon), C19 (rectosigmoid) and C20 (rectum), in the Netherlands from 1 January 2026 through 31 December 2022 were obtained from the NCR, a nationwide, population-based registry that captures data on all newly diagnosed malignancies in the Netherlands. Notification of malignancies is obtained through the Dutch nationwide pathology data network (Palga) and the National Registry of Hospital Discharge Diagnoses. Subsequently, trained NCR data administrators routinely collect data from electronic patient records.

Data entered into the NCR are comprehensively checked for possible registration errors, inconsistencies and duplicates. The overall completeness of case ascertainment of the NCR exceeds 95%.^21^ This study received approval from the scientific committee of the NCR (approval number 24-00327). Based on the regulations of the Central Committee on Research involving Human Subjects in the Netherlands, no additional approval from a Medical Research Ethics Committee was required.

The following data were retrieved: age at diagnosis, sex, pathological tumor-node-metastases (pTNM) stage (if pTNM was not available clinical (c)TNM stage was used), primary tumor location (left-sided colon (lienalis to sigmoid), right-sided colon (coecum to transversum), colon not otherwise specified (NOS), rectosigmoid and rectum), differentiation grade (well-moderate, poor), received treatment (including surgery, radiotherapy, chemoradiotherapy and systemic treatment administered as neoadjuvant, adjuvant or first-line palliative treatment), mismatch repair (MMR) status (MMR proficient (pMMR), MMR deficient (dMMR)), mutation status (*BRAF* and *RAS*), treatment response (classified as complete response [CR], partial response [PR], stable disease [SD] and progressive disease [PD]). For patients with multiple tumors, we selected the CRC with the highest TNM stage for subsequent analyses. Follow-up of vital status was available until February 1st 2024 through an annual linkage of the NCR to the National Municipal Personal Records Database.

### Ethics

This retrospective study was conducted with pseudonymized and aggregated data from the NCR, maintained by the Netherlands Comprehensive Cancer Organization (IKNL), approval number 24-00327. The NCR uses an opt-out approach to consent. Data release by IKNL is subject to strict regulations, with oversight provided by a Privacy Review Board and a scientific committee. According to Dutch legislation, this study does not fall under the scope of the Medical Research Involving Human Subjects Acts (WMO) and therefore did not require approval from a medical ethics review committee. The study was conducted in accordance with the Declaration of Helsinki.

### Matching

To investigate differences in survival between patients with pMMR eoCRC, aoCRC, and ooCRC, each eoCRC patient was matched with an aoCRC and an ooCRC patient.

Matching of the data was performed by the data scientist of the NCR using Statistical Analysis System (SAS), through the following steps: patients with a pMMR adenocarcinoma diagnosed from 1 January 2016 through 31 December 2022 and ≥18 years of age were included. Next, for each patient in the eoCRC group, one aoCRC patient and one ooCRC patient was selected with the same clinical characteristics. Searching for a match, the following match variables were used in running order:

1. Tumor stage (stage I, II, II, IV), tumor sidedness (left-sided (lienalis to sigmoid), right-sided (coecum to transversum), colon NOS, rectosigmoid and rectum), MMR status, *RAS* mutation present, *BRAF* mutation present), surgery on metastases performed, radiotherapy on metastases performed, and systemic agents.
2. All match variables above excluding systemic agents
3. All match variables above excluding systemic agents and *BRAF* mutation
4. A very limited set: stage (stage I, II, III, IV), tumor sidedness (left-sided, right-sided, rectosigmoid, rectum, unknown), organ surgery performed, radiotherapy (yes/no), chemotherapy (yes/no), target therapy (yes/no).

Systemic therapy was matched based in different groups of agents: capecitabine, 5-fluorouracil, tegafur/gimeracil/oteracil, tegafur; oxaliplatin; bevacizumab; irinotecan; panitumumab, cetuximab; trifluridine/tipiracil; nivolumab, ipilimumab, pembrolizumab, durvalumab, atezolizumab, tremelimumab; encorafenib; lapatinib, trastuzumab, pertuzumab, trastuzumab.

### Statistical analyses

#### Clinical characteristics

Baseline characteristics were summarized separately for eoCRC, aoCRC and ooCRC patients. Age at diagnosis, defined as eoCRC, aoCRC and ooCRC, sex, tumor location (left-sided colon, right-sided colon rectosigmoid, rectum and colon NOS), tumor stage (I-IV), MMR) status (pMMR and dMMR), *RAS/BRAF* mutation status (wild type, mutant), surgical resection of primary tumor (yes/no), local treatment of metastases (surgery/ablation and/or radiotherapy), and receiving (neo)adjuvant or palliative systemic therapy were included as categorical variables.

Categorical variables were reported as counts and percentages, and continuous variables as medians with interquartile ranges (IQR). To quantify differences in baseline characteristics between age groups, we calculated standardized mean differences (SMDs) for all variables. An absolute SMD <0·10 was considered to indicate negligible imbalance.

#### Survival and multivariable analyses

To estimate age-specific 5-year survival and to evaluate whether the age cut-off of 50 years for defining eoCRC was appropriate, a Cox proportional hazards model with restricted cubic splines for age at diagnosis using five knots placed at default locations (the 0·05, 0·275, 0·50, 0·725, and 0·95 quantiles of age distribution) was fitted as implemented in the *rcs()* function of the *rms* R package. We additionally modeled non-linear effects of age and their interaction with tumor stage by including an interaction between these spline terms and stage in the model. From this model, we estimated the survival at 5 years of follow-up for an individual of age 25 to 85 and plotted these estimates, including 95% CIs. For the adjusted analyses, we fitted a Cox proportional hazards model with restricted cubic splines for age at diagnosis with five knots, sex, tumor stage, tumor location, MMR status, grade of differentiation, and treatment as covariates. We then estimated the survival at 5 years follow-up for an individual of age 25 to 85 for stages I to IV, but with the following ‘set’ covariates: male, right-sided tumor location, pMMR, well/moderately differentiated, and surgery. These covariates were chosen as they were the most common, and because they were both a. not of scientific interest and b. less influential than age and stage. We again plotted the results with 95% CIs. The proportional hazards assumption for this model was assessed using scaled Schoenfeld residuals with an identity transformation. Given the relatively large sample size, we inspected the stability of covariate effects over time rather than relying solely on formal hypothesis testing and judged the average coefficients to be sufficiently stable to support the proportional hazards assumption. Scaled Schoenfeld residuals of all the separate covariate effects are reported in the Supplementary Material.

Overall survival (OS) was defined as the time from CRC diagnosis to the date of death from any cause; patients alive at last follow-up were censored at the last date.

Cox proportional hazards models estimated hazard ratios (HRs) and 95% confidence intervals (CIs) for the association between age group and OS. Relative survival (RS) was estimated using national life tables from Statistics Netherlands (CBS) as reference, following the Hakulinen estimator. Expected hazards were derived from population mortality rates stratified by age, sex, and calendar year. To compare excess mortality between age groups, univariable additive relative survival models were fitted using “rsadd” in “relsurv” (R software, version 4·3·2; R Core Team, Vienna, Austria) with age group (eoCRC, aoCRC and ooCRC) as the only covariate. These models yielded crude excess hazard ratios (EHRs) with 95% CIs, quantifying age-related differences in excess mortality above background population mortality.

A multivariable Cox proportional hazards regression model was used to examine associations between independent variables (including age, sex, tumor location, grade of differentiation, number of metastatic sites, *RAS* and *BRAF* mutation status, and treatment) and time to event. The proportional hazards assumption was evaluated as described above.

Data on follow-up, death or latest follow-up date, and age were complete. For multivariable analyses, missing values in categorical variables were included as a separate category (‘missing/unknown’). To assess the impact of missing data on survival estimates, sensitivity analyses were performed using complete cases and single imputation for the age specific estimates. Results were reported as HR or EHR with 95% CI. Statistical significance was defined as a two-sided *p*-value of *p* < 0·050. All analyses were performed with R software (version 4·3·2; R Core Team, Vienna, Austria). The following R packages were used: “tidyverse” (v 2·0·0) for data manipulation and visualization, “survival” (v 3·8·6) and “relsurv” (v 2·3·3) for survival analyses, “rms” (v 8·1·1) for regression modelling strategies, “tableone” (v 0·13·2) for baseline characteristics and standardized mean differences, “survminer” (v 0·5·2) and “ggsurvfit” (v 1·2·0) for visualization of survival curves, “gtsummary” (v 2·5·0) for table formatting, “forestplot” (v 3·2·0) for forest plots, and “officer” (v 0·7·3) and “openxlsx” (v 4·2·8·1) for export of results.

### Role of the funding source

The funder of the study had no role in the study design, data collection, data analysis, data interpretation, or writing of the report.

## Results

### Establishing the age threshold of eoCRC

In total, 80,551 patients were diagnosed with CRC from 1 January 2016 through 31 December 2022 in the Netherlands. We first evaluated whether the commonly used age cut-off of 50 years for defining eoCRC was appropriate in this cohort, or whether a lower age cut-off would be more appropriate. As displayed in Fig. 1A, the proportion of patients alive after 5 years increased with age up to approximately 60–65 years, after which it decreased. No distinct age-related cut-off associated with a worse prognosis was observed below the age of 50 years, indicating that lowering the age cut-off for defining eoCRC could not be supported. When stratified by tumor stage I-III (Fig. 1B), the proportion of patients alive after 5 years progressively decreased with increasing age. Among patients with stage IV disease at diagnosis, 5-year survival declined continuously, which became steeper from the age of 55 years onwards (Fig. 1B). Based on these findings, we retained the clinical definition of eoCRC as diagnosis before the age of 50 years for all subsequent analyses. To evaluate the impact of missing data on survival, we repeated the analyses in complete cases and using single imputation for missing covariates. The resulting age-specific estimate survival curves were highly similar to those of the primary analysis (Supplementary Figure S1). Therefore, all subsequent analyses were handled by coding missing covariate data as a separate ‘unknown’ category.

**Fig. 1:**
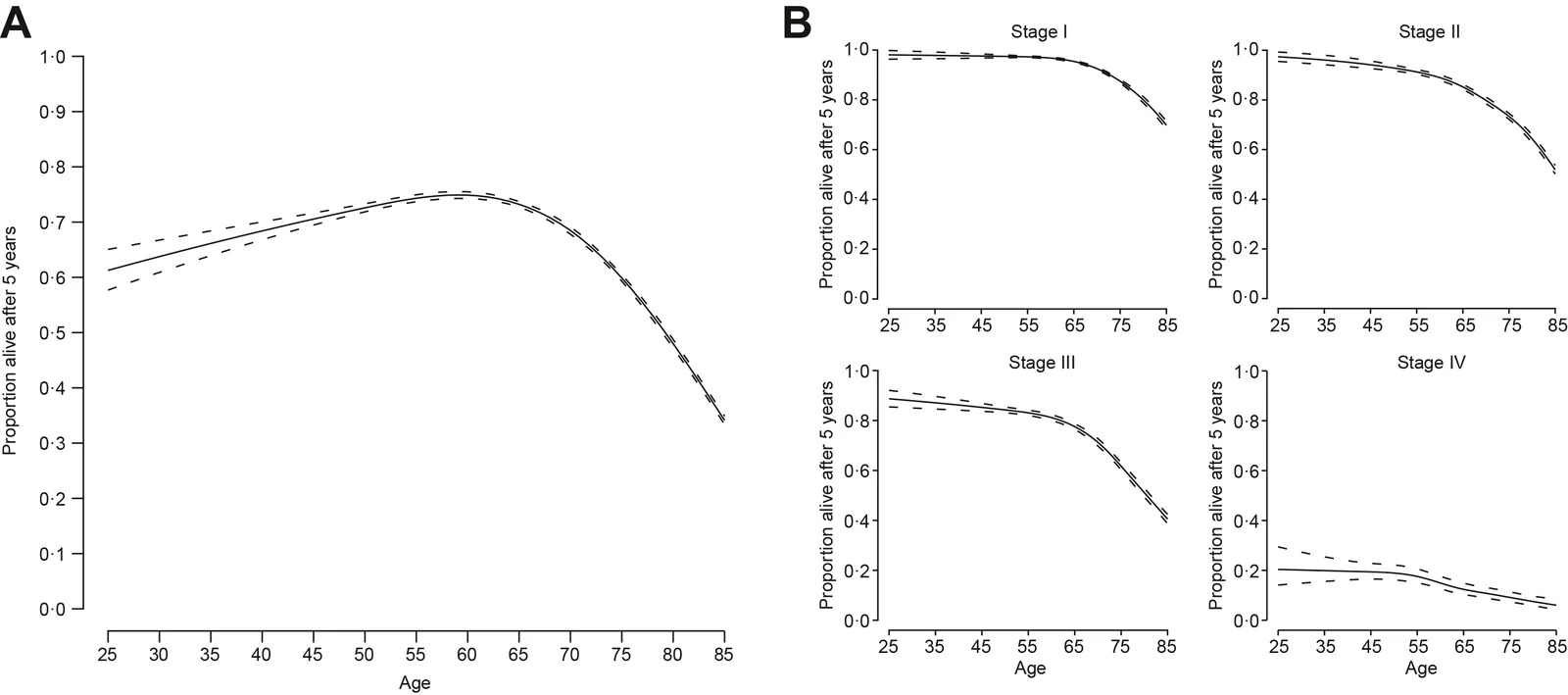
**Cox proportional hazards model for age-specific estimates of 5-year survival**. Age-specific estimates of survival at 5 years of follow-up, including 95% CIs, for an individual of age 25 to 85 are plotted. A Cox proportional hazards model with restricted cubic splines for age at diagnosis was fitted as a crude model. For the adjusted model, we also added sex, tumor stage, tumor location, MMR status, grade of differentiation, and treatment as covariates. **A.** Crude estimates of 5-year survival for all disease stages (I-IV) combined. **B.** Adjusted estimates of 5-year survival of patients with stage I, II, III, or IV at diagnosis. X-axis: age at diagnosis of CRC; Y axis: proportion of patients alive after 5-years. Abbreviations: CRC: colorectal cancer; CI: confidence interval.

### EoCRC characteristics and survival

Baseline characteristics of eoCRC patients are summarized in Table 1 and Supplementary Table S1. Of all patients with CRC, 3917 (5%) were diagnosed below the age of 50 years (eoCRC), with a median age of 45 (IQR 41–48) years. MMR status of the tumor was known in 3337 (85%) eoCRC patients, of whom 3033 (91%) had pMMR tumors (Table 1). Among pMMR eoCRC patients, the primary tumor was more frequently located in the left-sided colon or rectum than among those with dMMR tumors (78% vs. 40% in dMMR; SMD = 0·91). Patients with pMMR eoCRC more frequently presented with metastatic disease at diagnosis than patients with dMMR eoCRC (31% (n = 956) vs. 14% (n = 42) in dMMR; SMD = 0·51). Consequently, a higher proportion of pMMR eoCRC patients received systemic therapy compared to patients with dMMR eoCRC (Supplementary Table S1).

**Table 1:** Clinicopathological characteristics of all patients with CRC diagnosed from 1 January 2016 through 31 December 2022 in the Netherlands.

	eoCRC (n = 3917) n (%)	aoCRC (n = 34,499) n (%)	ooCRC (n = 42,135) n (%)	SMD^b^ <50 vs. 50–69	SMD^b^ 50–69 vs. ≥ 70
Age (years)					
Median (IQR)	45 (41–48)	62 (57–66)	77 (73–82)		
Sex				0·12	0·05
Male	2015 (51)	19,832 (58)	23,097 (55)		
Female	1902 (49)	14,667 (42)	19,038 (45)		
Tumor location				0·13	0·30
Right colon	861 (22)	9474 (28)	17,292 (41)		
Left colon	1465 (37)	12,213 (35)	12,631 (30)		
Rectosigmoid	123 (3)	1092 (3)	1063 (2)		
Rectum	1425 (37)	11,294 (33)	10,463 (25)		
Colon NOS	43 (1)	426 (1)	686 (2)		
MMR status^c^				0·03^a^	0·28^a^
dMMR	304 (9)	2286 (8)	2147 (18)		
pMMR	3033 (91)	24,908 (92)	9859 (82)		
Unknown	580	7305	30,131		
Grade of differentiation^c^				0·09^a^	0·09^a^
Well/moderate	3181 (89)	29,205 (92)	34,202 (89)		
Poor	384 (11)	2553 (8)	4113 (11)		
Unknown	352	2741	3820		
Stage^c^				0·46^a^	0·19^a^
Stage I	539 (14)	10,557 (31)	10,923 (27)		
Stage II	687 (18)	6617 (19)	11,225 (27)		
Stage III	1501 (38)	10,493 (31)	11,376 (28)		
Stage IV	1179 (30)	6521 (19)	7536 (18)		
Unknown	11	311	1075		
Surgical resection (stage I-III only)^d^	**n** = **2727**	**n** = **27,667**	**n** = **33,524**	0·14	0·06
Yes	2414 (88)	23,174 (84)	27,354 (82)		
No	313 (12)	4493 (16)	6170 (18)		
Treatment (stage I-III only)^d^	**n** = **2727**	**n** = **27,667**	**n** = **33,524**	0·49	0·46
Surgery alone	958 (35)	13,606 (49)	20,998 (63)		
(Neo)adjuvant chemotherapy + CRT ( ± surgery)	420 (15)	2281 (8)	1242 (4)		
(Neo)adjuvant chemotherapy + RT ( ± surgery)	31 (1)	117 (<0·5)	44 (<0·5)		
(Neo)adjuvant CRT ± surgery	131 (5)	925 (3)	675 (2)		
(Neo)adjuvant RT ± surgery	143 (5)	1718 (6)	2554 (8)		
(Neo)adjuvant chemotherapy + surgery	873 (32)	5583 (20)	3538 (11)		
Systemic therapy without surgery	35 (1)	154 (1)	164 (<0·5)		
Other	7 (<0·5)	25 (<0·5)	10 (<0·5)		
No treatment	2 (<0·5)	190 (1)	1437 (4)		
Unknown	127 (5)	3068 (11)	2862 (9)		

Abbreviations: CRC; colorectal cancer; eoCRC: early-onset colorectal cancer; aoCRC: average-onset colorectal cancer; ooCRC: older-onset colorectal cancer SMD: standard mean difference; IQR: interquartile range; NOS: not otherwise specified; MMR: mismatch repair; dMMR: deficient mismatch repair; pMMR: proficient mismatch repair; CRT: chemoradiotherapy; RT: radiotherapy.

Data are n (%).

a SMD was assessed excluding cases with unknown values.

b SMD values closer to 0 indicate smaller differences between age groups.

c Data not available for all patients.

d Treatment showed for only stage I-III, stage IV is shown in Table 2.

As shown in Supplementary Figure S2, RS of eoCRC patients with a dMMR tumor, across all disease stages, was significantly better than that of patients with a pMMR tumor, with a 5-year RS of 91% and 68% respectively (EHR, 0·25 [95% CI 0·17–0·38]; Supplementary Figure S2A). Among eoCRC patients with pMMR disease stage I, II, III, and IV, 5-year RS was 97%, 93%, 87% and 20%, respectively (Supplementary Figure S2B). Within metastatic eoCRC, patients with a right-sided *BRAF*-mutated cancer had the poorest RS, whereas those with left-sided *BRAF*/*RAS* wild type tumors had the most favorable outcomes, with median relative survivals of 9 and 29 months, respectively (Supplementary Figure S3).

### Comparison of characteristics and survival of eoCRC, aoCRC and ooCRC patients

We next compared clinical characteristics and survival outcomes of eoCRC patients with two older age groups: patients aged 50–69 years (aoCRC; n = 34,499 [43%], median age 62 years [IQR 57–66]) and patients aged 70 years or older (ooCRC; n = 42,135 [52%], median age 77 years [IQR 73–82]) at diagnosis. Clinical characteristics and survival outcomes are shown in Table 1 and Fig. 2 dMMR tumors were more prevalent in ooCRC patients (vs. aoCRC, SMD = 0·28). Left-sided and rectal tumors were more commonly diagnosed in eoCRC (74%, n = 2890) and aoCRC (68%, n = 23,507) patients compared to ooCRC patients (55%, n = 23,094). EoCRC patients were more frequently diagnosed with advanced disease (stage III or IV) than aoCRC and ooCRC patients (vs. aoCRC, SMD = 0·46; vs. ooCRC, SMD = 0·48; Table 1).

**Fig. 2:**
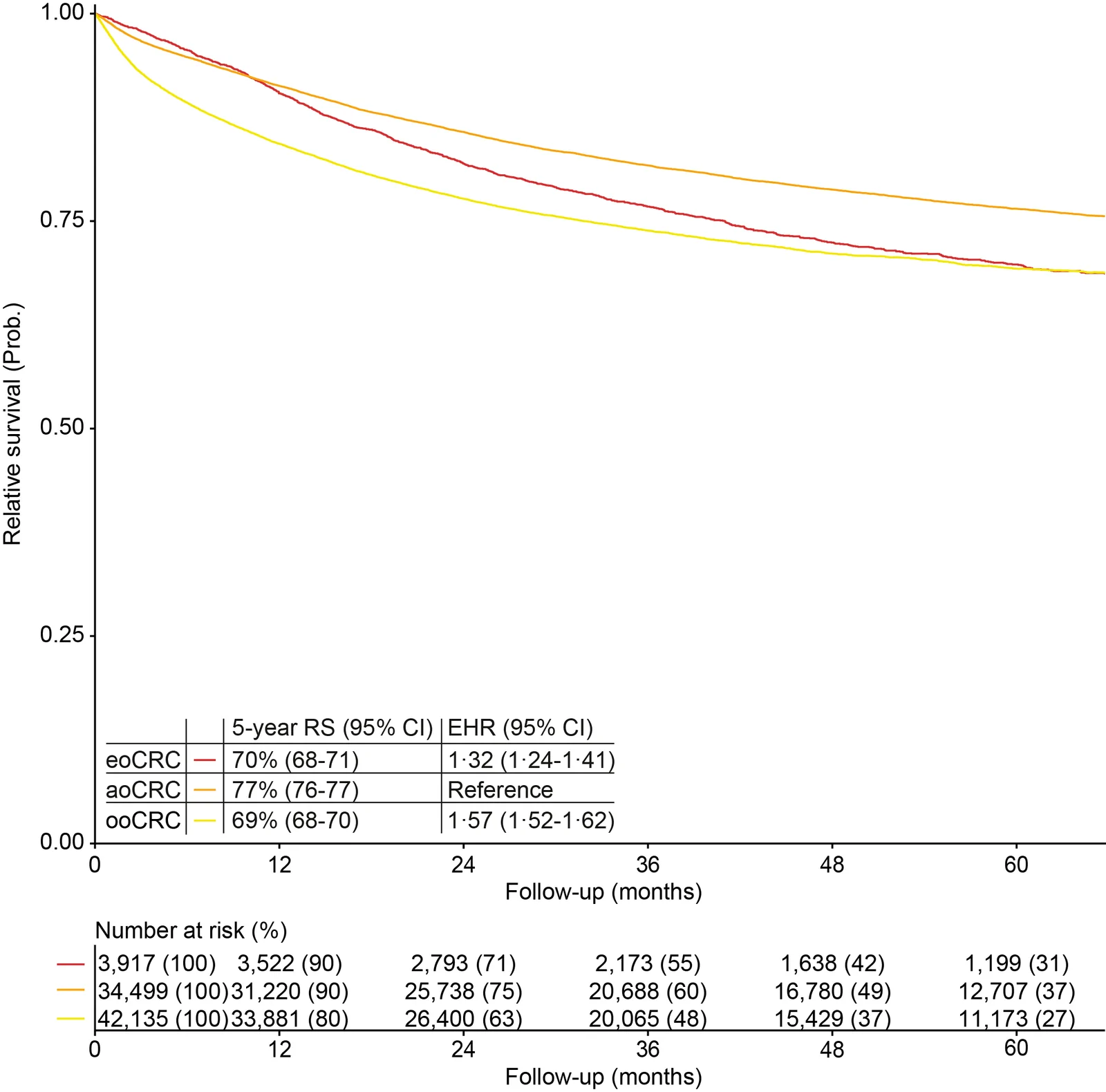
**Relative survival of all patients with CRC included in this cohort**. Kaplan–Meier RS curve of all patients included in this cohort, stratified by three age groups, eoCRC, aoCRC and ooCRC. Crude EHRs for RS were estimated using univariable additive relative survival models with age groups as the only covariate, again using aoCRC as the reference. Abbreviations: CRC: colorectal cancer; RS: relative survival; eoCRC: early-onset colorectal cancer; aoCRC: average-onset colorectal cancer; ooCRC: older-onset colorectal cancer; EHR: excess hazard ratio; CI: confidence interval.

As shown in Fig. 2, five-year RS rates of aoCRC patients were better than eoCRC and ooCRC patients, at 70%, 77% and 69% for eoCRC, aoCRC and ooCRC patients, respectively.

### (Neo)adjuvant treatment and survival of eoCRC, aoCRC and ooCRC patients

To further investigate differences in whether or not patients with colon cancer received (neo)adjuvant chemotherapy and whether outcomes differed, we analyzed patients with high-risk stage II and stage III colon cancer (excluding rectal cancer), with an indication for adjuvant chemotherapy. As displayed in Supplementary Table S2, 2010 (2·5%) were diagnosed with high-risk stage II (T4N0M0) colon cancer, and 13,829 (17%) were diagnosed with stage III colon cancer. In total, 583 patients (combined eoCRC, aoCRC and ooCRC patients) with high-risk stage II CRC (29%), 6239 patients (combined eoCRC, aoCRC and ooCRC patients) with low-risk stage III CRC (65%) and 2831 patients (combined eoCRC, aoCRC and ooCRC patients) with high-risk stage III CRC (68%) received (neo)adjuvant chemotherapy, mainly consisting of doublet systemic therapy (capecitabine-oxaliplatin/5-fluorouracil-leucovorin-oxaliplatin [CAPOX/FOLFOX]) in all age groups. OoCRC patients received (neo)adjuvant systemic therapy less frequently than aoCRC patients (high-risk stage II, SMD = 0·99; low-risk stage III, SMD = 1·07, high-risk stage III, SMD = 1·19).

RS among patients with high-risk stage II and high- and low-risk stage III disease is shown in Supplementary Figure S4. RS of eoCRC patients was comparable to that of aoCRC patients (EHR, 0·79 [95% CI 0·33–1·84] for high-risk stage II; EHR, 1·30 [95% CI 0·92–1·84] for low-risk stage III; EHR, 0·80 [95% CI 0·60–1·07] for high-risk stage III). RS of ooCRC patients with high-risk stage III was significantly worse compared to aoCRC (EHR, 1·39 [95% CI 1·19–1·62]). Total neoadjuvant treatment (TNT), consisting of chemotherapy combined with chemoradiotherapy (CRT) or radiotherapy (RT) is increasingly used in treatment of patients with locally advanced rectal cancer. In total, 4135 (28%) patients (94% rectal cancer) received TNT. EoCRC patients more often received TNT compared to aoCRC and ooCRC patients (16% (n = 451) vs. 8% (n = 2398) and 4% (n = 1286); vs. aoCRC, SMD = 0·49, vs. ooCRC, SMD = 0·90; Table 1).

### Disease characteristics, treatment and outcome of eoCRC, aoCRC and ooCRC patients with metastatic pMMR CRC at diagnosis

We next evaluated treatment patterns and outcome among patients diagnosed with pMMR metastatic CRC. Clinical and treatment characteristics of eoCRC, aoCRC and ooCRC patients are shown in Table 2 and Fig. 3A–C. Overall, 787 (82%) eoCRC, 3549 (72%) aoCRC, and 1513 (60%) ooCRC patients received systemic therapy at diagnosis of stage IV pMMR CRC. The proportion of patients who did not receive any local treatment was 61% (n = 586), 66% (n = 3225) and 79% (n = 1992) for eoCRC, aoCRC, and ooCRC. EoCRC patients more often received first-line triplet therapy (5-fluorouracil-leucovorin-oxaliplatin-irinotecan [FOLFOXIRI] ± bevacizumab) than aoCRC and ooCRC patients (*p* < 0·0001; 20% (n = 161), 10% (n = 342) and 3% (n = 39), respectively; Fig. 3B). In contrast, ooCRC patients more often received fluoropyrimidine monotherapy ± bevacizumab (*p* < 0·0001; 8% (n = 64), 14% (n = 487), and 40% (n = 599), respectively; Fig. 3B). Best response to first-line systemic therapy was more favorable in eoCRC and aoCRC patients than in ooCRC patients (*p* < 0·0001; Fig. 3C).

**Table 2:** Clinical characteristics of patients with stage IV pMMR CRC at diagnosis.

	eoCRC (n = 956) n (%)	aoCRC (n = 4904) n (%)	ooCRC (n = 2533) n (%)	SMD^d^ <50 vs. 50–69	SMD^d^ 50–69 vs. ≥70
Sex				0·14	0·03
Male	490 (51)	2855 (58)	1509 (59)		
Female	466 (49)	2049 (42)	1026 (41)		
Tumor location				0·20	0·28
Right colon	213 (22)	1492 (30)	1071 (42)		
Left colon	369 (39)	1743 (35)	813 (32)		
Rectosigmoid	32 (3)	133 (3)	44 (2)		
Rectum	331 (35)	1454 (30)	538 (21)		
Colon NOS	11 (1)	82 (2)	67 (3)		
Grade of differentiation^e^				0·10^a^	0·06^a^
Well/moderate	661 (81)	3511 (85)	1667 (83)		
Poor	152 (19)	624 (15)	352 (17)		
Unknown	143	769	514		
Number of metastatic sites^e^				0·07^a^	0·03^a^
One	543 (57)	2759 (56)	1419 (56)		
Liver	402 (74)	1998 (72)	992 (70)		
Lung	31 (6)	238 (9)	139 (10)		
Lymph nodes	46 (9)	195 (7)	95 (6)		
Peritoneal	51 (9)	293 (10)	172 (12)		
Other	13 (2)	44 (2)	23 (2)		
Two	240 (25)	1353 (28)	723 (29)		
Three or more	173 (18)	784 (16)	387 (15)		
Unknown	0	8	4		
*RAS*^e^				0·04^a^	0·07^a^
Wild type	321 (48)	1318 (46)	656 (43)		
Mutation	341 (52)	1521 (54)	862 (57)		
Unknown^f^	294	2065	1015		
*BRAF*^e^				0·11^a^	0·015^a^
Wild type	532 (89)	2215 (85)	1191 (86)		
Mutation	67 (11)	390 (15)	201 (14)		
Unknown^f^	357	2299	1141		
Local treatment				0·09	0·30
Surgery/ablation metastases	324 (34)	1476 (30)	447 (18)		
Radiotherapy metastases	33 (3)	145 (3)	74 (3)		
Surgery + radiotherapy metastases	13 (1)	58 (1)	20 (1)		
No local treatment	586 (61)	3225 (66)	1992 (79)		
Systemic treatment^b^				0·39	0·60
Fluoropyrimidine monotherapy^c^	64 (7)	487 (10)	599 (24)		
FOLFOX/CAPOX	511 (53)	2561 (52)	833 (33)		
FOLFIRI/CAPIRI	17 (2)	59 (1)	16 (1)		
FOLFOXIRI/CAPOXIRI	161 (17)	342 (7)	39 (2)		
Anti-*EGFR* therapy^g^	25 (3)	75 (2)	11 (<05)		
Other	9 (1)	25 (1)	15 (1)		
No systemic therapy	169 (18)	1355 (28)	1020 (40)		

Data are n (%).

Abbreviations: CRC: colorectal cancer; eoCRC: early-onset colorectal cancer; aoCRC: average-onset colorectal cancer; ooCRC: older-onset colorectal cancer; pMMR: proficient mismatch repair; IQR: interquartile range; NOS: not otherwise specified; 5-FU: 5-fluorouracil; FOLFOX: 5-fluorouracil-leucovorin-oxaliplatin; CAPOX: capecitabine-oxaliplatin; FOLFIRI: 5-fluorouracil-leucovorin-irinotecan; CAPIRI: capecitabine-irinotecan; FOLFOXIRI: 5-fluorouracil-leucovorin-oxaliplatin-irinotecan; CAPOXIRI; capecitabine-oxaliplatin-irinotecan; *EGFR:* epidermal growth factor receptor.

a SMD was assessed excluding cases with unknown values.

b all systemic treatments are ± bevacizumab, except for anti-*EGFR* therapy.

c 5-FU/capecitabine/tegafur-gimeracil-oteracil.

d SMD values closer to 0 indicate smaller differences between age groups.

e Data not available for all patients.

f For RAS, 31%, 42%, and 40% of eoCRC, aoCRC, and ooCRC was missing. For BRAF, 37%, 47%, and 45% of eoCRC, aoCRC, and ooCRC was missing.

g Anti-*EGFR* containing therapy (monotherapy or in combination with chemotherapy).

**Fig. 3:**
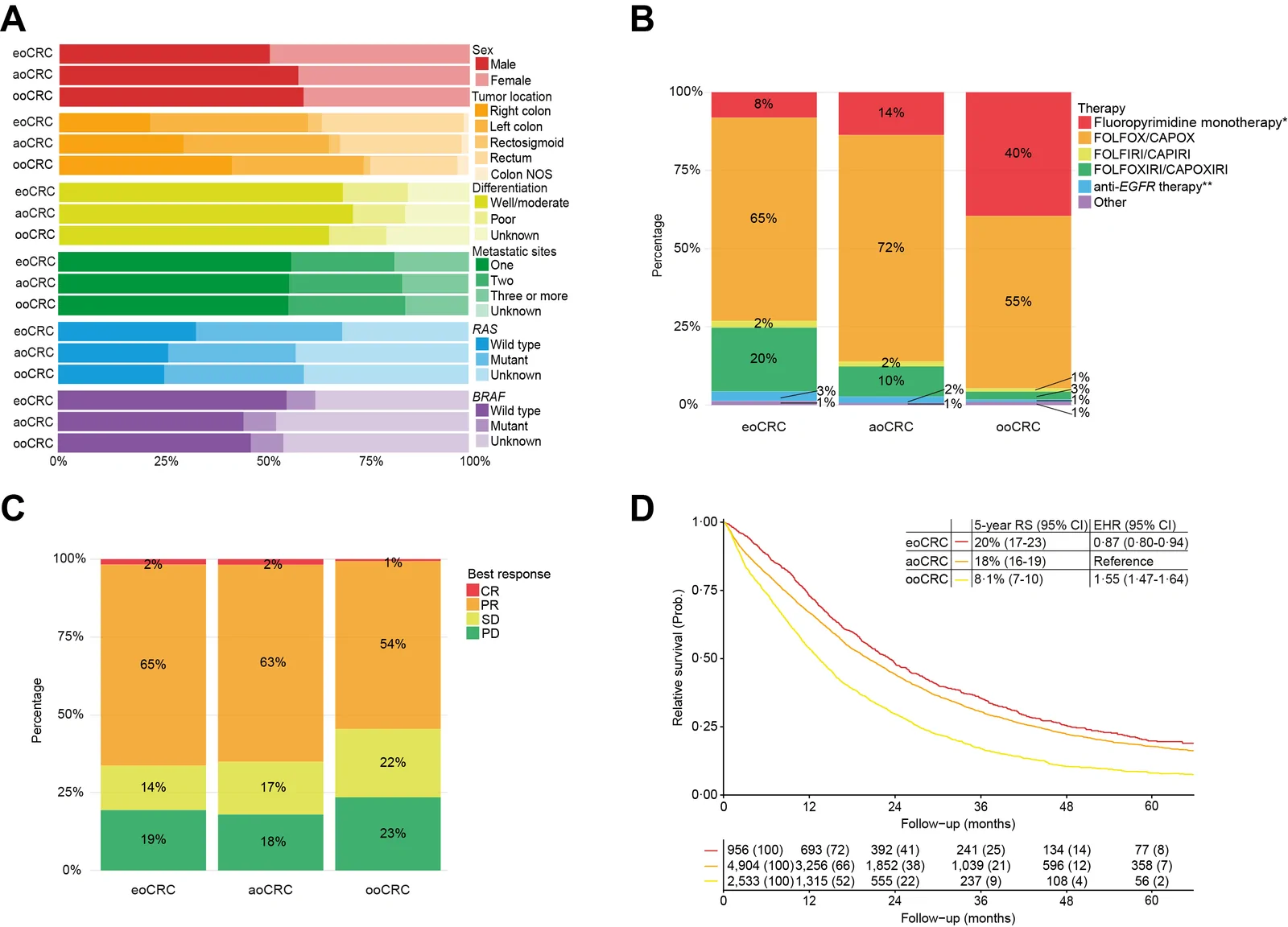
**Differences in treatment and outcome of eoCRC, aoCRC and ooCRC patients with stage IV pMMR CRC**. **A.** Clinical characteristics of patients with stage IV pMMR CRC for the three age categories (eoCRC, aoCRC, and ooCRC). **B.** Percentage of patients that received monotherapy∗, doublet therapy, triplet therapy, all ± bevacizumab, or anti-*EGFR* containing therapy∗∗ in the first-line. Percentages are calculated among patients who received systemic therapy (patients without systemic therapy are not included in this analysis). **C.** Best response to first-line therapy for the different age groups, defined as CR, PR, SD, or PD. **D**. Kaplan–Meier RS curve of patients with stage IV pMMR CRC stratified by three age groups, eoCRC, aoCRC and ooCRC. Crude EHRs for RS were estimated using univariable additive relative survival models with age groups as the only covariate, aoCRC patients served as the reference category. ∗Monotherapy: 5-FU/capecitabine/tegafur-gimeracil-oteracil. ∗∗Anti-*EGFR* containing therapy (monotherapy or in combination with chemotherapy). Abbreviations: eoCRC: early-onset colorectal cancer; aoCRC: average-onset colorectal cancer; ooCRC: older-onset colorectal cancer; pMMR: proficient mismatch repair; RS: relative survival; EHR: excess hazard ratio; NOS: not otherwise specified; CR: complete response; PR: partial response; SD: stable disease; PD: progressive disease; CI: confidence interval; 5-FU: 5-fluorouracil; CAPOX: capecitabine-oxaliplatin; FOLFOX: 5-fluorouracil-leucovorin-oxaliplatin; FOLFIRI: 5-fluorouracil-leucovorin-irinotecan; CAPIRI: capecitabine-irinotecan; FOLFOXIRI: 5-fluorouracil-leucovorin-oxaliplatin-irinotecan; CAPOXIRI; capecitabine-oxaliplatin-irinotecan. *EGFR*: epidermal growth factor receptor.

As shown in Fig. 3D, among patients with stage IV pMMR CRC, eoCRC patients had better RS than aoCRC patients (EHR 0·87 95% CI 0·80–0·94), whereas RS of ooCRC patients was significantly worse (vs. eoCRC: EHR 1·78 [95% CI 1·64–1·95]; vs. aoCRC: EHR 1·55 [95% CI 1·47–1·64]). Multivariable Cox proportional hazards regression analysis showed that primary tumor location, grade of differentiation, number of metastatic sites, and *BRAF/RAS* mutation status were independently associated with relative survival (Fig. 4). Triplet therapy (FOLFOXIRI or capecitabine-oxaliplatin-irinotecan [CAPOXIRI] ± bevacizumab) was associated with improved RS compared with doublet therapy (FOLFOX/CAPOX ± bevacizumab) in eoCRC and aoCRC patients (EHR 0·71 [95% CI 0·57–0·89] and EHR 0·79 [95% CI 0·68–0·93], respectively). In ooCRC patients, fluoropyrimidine monotherapy (5-FU/capecitabine/tegafur-gimeracil-oteracil ± bevacizumab) yielded an EHR of 1·12 [95% CI 0·99–1·27] compared with FOLFOX/CAPOX. To complement these analyses, Kaplan–Meier RS curves were generated comparing, triplet, doublet and fluoropyrimidine monotherapy within each age group and across age groups (Supplementary Figures S5 and S6). Given the potential for considerable confounding by indication, these regimen-specific comparisons were only exploratory.

**Fig. 4:**
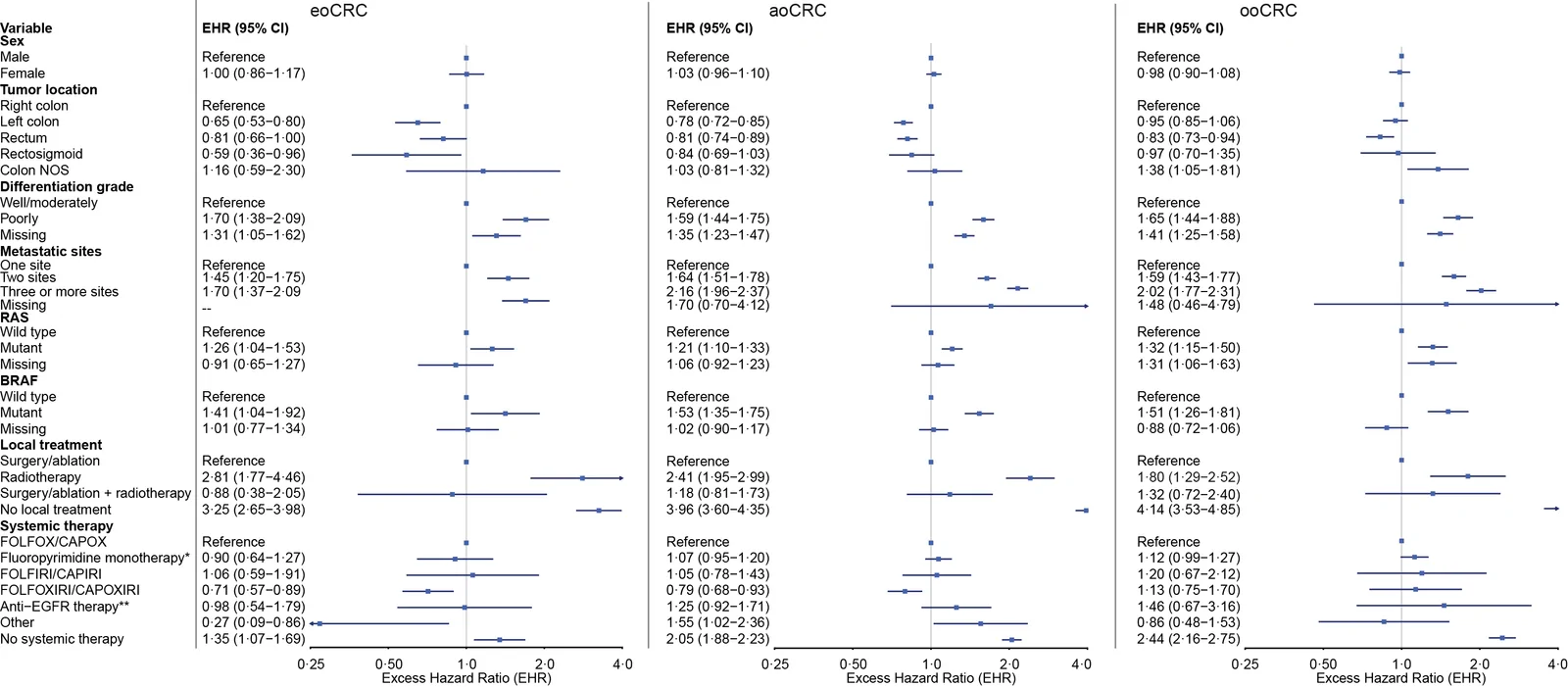
**Forest plot of adjusted EHRs for RS in patients with stage IV pMMR CRC, shown separately for three age groups (eoCRC, aoCRC and ooCRC)**. Within each age group, EHRs are derived from multivariable additive relative survival model including age, sex, tumor location (right colon, left colon, rectosigmoid, rectum, and colon NOS), differentiation (well/moderate, poor, unknown), number of metastatic sites (one, two, three or more, unknown), *RAS* mutation status (wild type, mutation, unknown), *BRAF* mutation status (wild type, mutation, unknown), local treatment for metastases (surgery/ablation, radiotherapy, combined surgery and radiotherapy, no local treatment), and systemic treatment (fluoropyrimidine monotherapy∗, FOLFOX/CAPOX, FOLFIRI/CAPIRI, FOLFOXIRI/CAPOXIRI, all ± bevacizumab, anti-*EGFR* containing therapy∗∗, other, no systemic therapy). Reference categories are male for sex, right colon for tumor location, well/moderate for tumor differentiation, one for number of metastatic sites, wild type for *RAS* mutation status, wild type for *BRAF* mutation status, surgery for local treatment, FOLFOX/CAPOX ( ± bevacizumab) for systemic treatment. ∗Monotherapy: 5-FU/capecitabine/tegafur-gimeracil-oteracil. ∗∗Anti-*EGFR* containing therapy (monotherapy or in combination with chemotherapy). Abbreviations: eoCRC: early-onset colorectal cancer; aoCRC: average-onset colorectal cancer; ooCRC: older-onset colorectal cancer; pMMR: proficient mismatch repair; RS: relative survival; EHR: excess hazard ratio; NOS: not otherwise specified; CR: complete response; PR: partial response; SD: stable disease; PD: progressive disease; CI: confidence interval; 5-FU: 5-fluorouracil; CAPOX: capecitabine-oxaliplatin; FOLFOX: 5-fluorouracil-leucovorin-oxaliplatin; FOLFIRI: 5-fluorouracil-leucovorin-irinotecan; CAPIRI: capecitabine-irinotecan; FOLFOXIRI: 5-fluorouracil-leucovorin-oxaliplatin-irinotecan; CAPOXIRI; capecitabine-oxaliplatin-irinotecan; *EGFR*: epidermal growth factor receptor.

### Comparison of eoCRC patients with matched aoCRC and ooCRC patients

To assess the extent to which clinicopathological characteristics and treatment explained survival differences, the three age groups were matched, resulting in a matched cohort of 2830 eoCRC, 2604 aoCRC, and 1995 ooCRC patients. Baseline characteristics of the matched cohorts are presented in Supplementary Tables S3 and S4. The median age was 45 years (IQR 41–48) for eoCRC, 61 years (IQR 56–65) for aoCRC and 74 years (IQR 71–77) for ooCRC patients.

In the matched cohort, as showed in Fig. 5, 5-year RS was 68%, 68% and 66% for eoCRC, aoCRC, and ooCRC patients, respectively (Fig. 5A). In contrast to the unmatched analyses, no significant difference in RS was observed between eoCRC and aoCRC patients, whereas RS remained significantly worse in ooCRC patients (EHR 1·15 [95% CI 1·02–1·31]). Similarly, among patients with stage IV disease at diagnosis, median RS was comparable between eoCRC and aoCRC patients (24 vs. 23 months) and significantly shorter in ooCRC patients (19 months; EHR 1·17 [95% CI 1·02–1·34], for ooCRC vs. aoCRC; Fig. 5B).

**Fig. 5:**
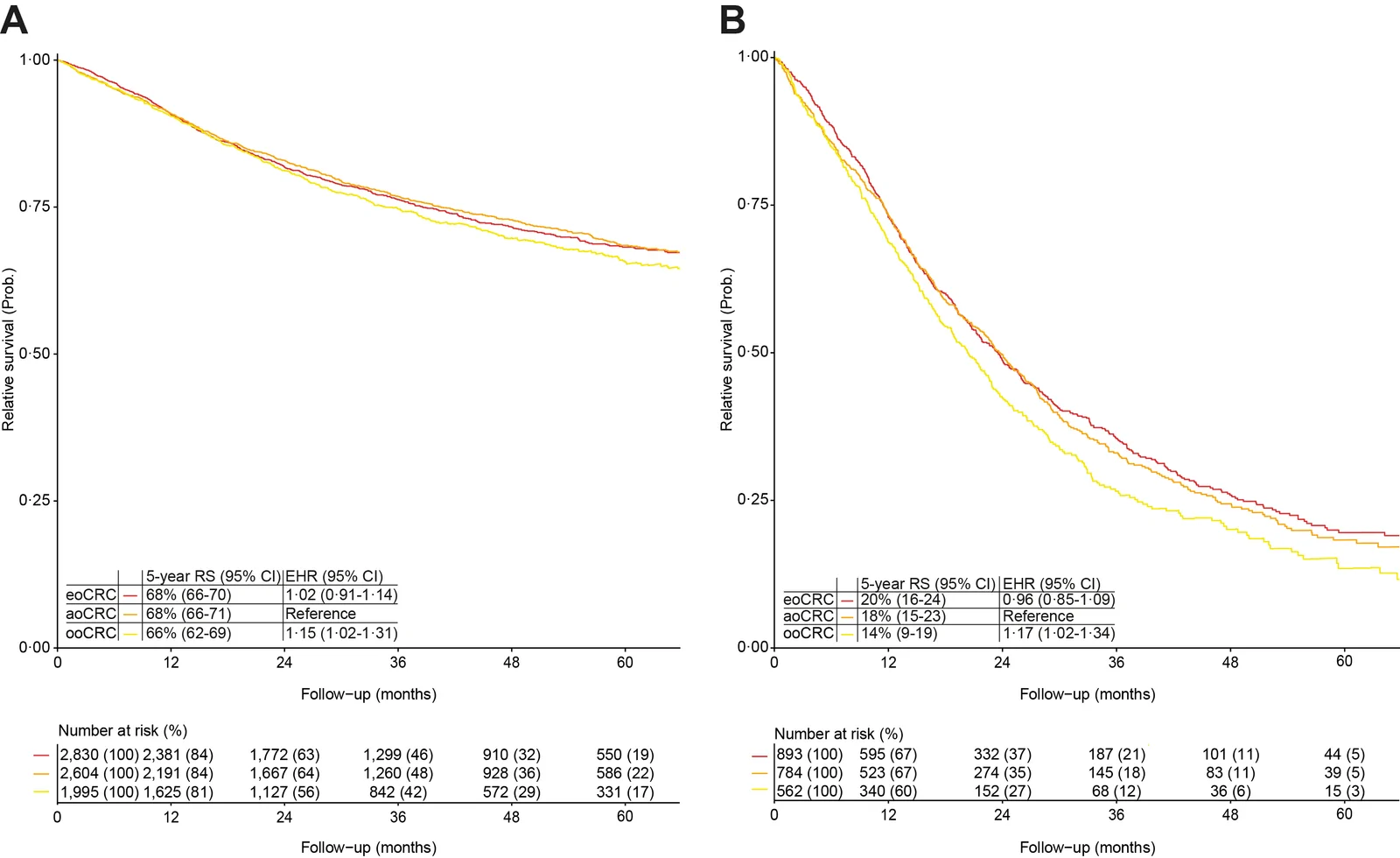
**Comparison of eoCRC patients with matched aoCRC and ooCRC patients**. **A.** Kaplan–Meier RS curves of patients with pMMR CRC of the three age groups (eoCRC, n = 2830; aoCRC, n = 2604; and ooCRC, n = 1995) matched on tumor stage, tumor sidedness, *BRAF* and *RAS* mutation status, and received treatment. Crude EHRs for RS were estimated using univariable additive relative survival models with age groups as the only covariate, aoCRC patients served as the reference category. **B.** Kaplan–Meier RS curves of patients with pMMR stage IV CRC stratified by the same age groups. Crude EHRs for RS were estimated using univariable additive relative survival models with age groups as the only covariate, again using aoCRC as the reference. Abbreviations: eoCRC: early-onset colorectal cancer; aoCRC: average-onset colorectal cancer; ooCRC: older-onset colorectal cancer; RS; relative survival; EHR; excess hazard ratio; pMMR; proficient mismatch repair; CI: confidence interval.

## Discussion

This nationwide, registry-based analysis shows that the poorer survival of eoCRC compared with aoCRC patients is largely explained by differences in stage at diagnosis, tumor characteristics, and treatment patterns; after matching for these factors, survival is comparable, whereas outcomes in ooCRC patients remain worse.

To examine the rationale underlying the clinically defined age cut-off of 50 years for eoCRC, we modeled age-specific estimates for 5-year survival. There was no noticeable change in the slope of the curve below the age of 50 years. We therefore concluded that there was no rationale for lowering the age threshold used to define eoCRC, and we support maintaining this age threshold for the definition of eoCRC. Survival increased up to the age of 60–65 years and declined thereafter, likely reflecting both shorter life expectancy in older patients and the impact of population-based FIT (fecal immunochemical test) screening from the age of 55 years in the Netherlands.^22^

EoCRC is often described as a more aggressive disease entity, less responsive to current treatments, and inferior outcomes, raising the question of whether it reflects a distinct tumor biology. However, eoCRC is more frequently diagnosed at an advanced stage, likely due to diagnostic delay and absence of routine screening, which may create the impression of greater aggressiveness rather than reflecting intrinsic biological differences.^9^, ^23^, ^24^ Consistent with this, eoCRC patients in our cohort more often presented with stage III or IV disease, and survival differences largely disappeared after matching for disease characteristics and treatment. These findings underscore that earlier detection and timely diagnostic work-up, rather than redefining age thresholds, are central to improving outcomes in eoCRC.

In contrast to some prior reports, we did not observe an overrepresentation of poor histological subtypes or aggressive molecular features, including *KRAS* mutations, in eoCRC.^12^, ^13^ Discrepant findings across studies may be partly explained by unmatched tumor characteristics and heterogeneous age definitions for eoCRC. Our data highlight substantial heterogeneity within eoCRC, patients with dMMR eoCRC have encouraging survival, whereas our principal conclusions apply primarily to the larger group of pMMR, presumably sporadic, eoCRC.

Among patients with high-risk stage III colon cancer, eoCRC patients had overall comparable RS to aoCRC patients, with a non-significant trend toward better RS in eoCRC. This finding contrasts with reports of worse recurrence-free survival in younger patients.^11^ Differences in age stratification and study design may account for this discrepancy.

In patients with stage IV pMMR CRC, eoCRC patients had better RS than aoCRC patients, despite similar rates of best response to first-line therapy. EoCRC patients more frequently received intensified systemic treatment regimens, raising the question whether improved survival reflects treatment intensity rather than tumor biology. Intensified therapy with FOLFOXIRI has demonstrated survival benefit in fit patients with metastatic CRC irrespective of age,^25^ and similar benefits were observed across age groups in our cohort. However, comparable response rates between eoCRC and aoCRC patients treated with standard doublet regimens argue against the routine need for treatment escalation solely based on young age. EoCRC patients not only receive intensified treatment in the metastatic setting, they also more often receive TNT for rectal cancer. Survival benefit of more intensified treatment in this population remains unclear.^26^ To explore regimen-specific survival, we compared triplet, doublet and monotherapy within and across age groups. However, real-world treatment selection depends strongly on clinical judgement and patient preferences. Since detailed data on performance status, tumor burden and location of metastases was not fully available, these associations face considerable confounding by indication and do not necessarily reflect causal treatment effects.

These findings add to growing concerns that eoCRC patients may be exposed to more intensive treatment than clinically required, without proportional survival benefit. Prior studies have shown that eoCRC patients are more likely to receive aggressive multimodality treatment, often without clear gains in outcome, raising the possibility of overtreatment.^19^, ^20^ Given the improving life expectancy of young patients, unnecessary treatment escalation may have important implications for long-term toxicity and quality of life. In the curative setting, intensive multimodal regimens carry risks of long-term toxicity without guaranteed survival benefit.^11^

Interpretation of survival differences in metastatic disease is limited by the absence of data on subsequent treatment lines, which are likely administered more frequently to eoCRC patients. Prior studies report inconsistent survival outcomes in eoCRC, and improved cancer-specific survival has been attributed to greater exposure to systemic therapy.^27^, ^28^ Together, these observations support the need to critically evaluate treatment intensity in eoCRC and to avoid extrapolating age alone as an indication for more aggressive therapy.

Consistent with previous studies, eoCRC patients were more frequently diagnosed with synchronous metastatic disease. Because only synchronous metastases were captured, potentially favorable outcomes associated with metachronous metastases, particularly liver-only disease, could not be assessed,^29^, ^30^, ^31^ contributing to heterogeneity across studies. Notably, incidence of metachronous metastases increases with more advanced stage at diagnosis (e.g. stage III).^31^ Since eoCRC patients are more frequently diagnosed with stage III disease compared to aoCRC and ooCRC patients, survival differences between eoCRC patients with synchronous and metachronous metastases could potentially be smaller than in aoCRC and ooCRC patients.

This study has several strengths, including the use of contemporary, nationwide data, a large eoCRC population, detailed treatment information and key molecular markers. Matching across age groups for multiple prognostic and predictive variables enabled a more direct comparison of outcomes than most previous reports. Limitations include the lack of data on comorbidities, frailty, performance status, sequential therapies and cancer-specific mortality. We therefore cannot rule out the possibility that fitter patients received more sequential lines of treatment or a higher cumulative chemotherapy dose, which may have influenced survival outcomes. Residual confounding by unmeasured molecular features beyond MMR status and *RAS*/*BRAF* mutations cannot be excluded. Although according to the national guidelines, *RAS/BRAF* mutations status must be determined when patients are treated with systemic therapy for metastatic CRC, we still observed missing values, as mutation testing may have been performed only in later treatment lines or not at all in routine practice. This reflects the real-world nature of our data. Consequently, such information is missing by design rather than at random, which challenges the standard missing at random assumption required for conventional multiple imputation models. Multiple imputation may introduce additional model dependence, with estimates becoming sensitive to the specific chosen imputation model. Additionally, we have missing data in other binary and categorical variables, which we included as a separate category in the analysis. Although this is crude and may suffer from the missing at random (MAR) principle, it is the most transparent approach, given that treatment and molecular characteristics are assessed in certain situations, which does not warrant imputation under MAR, whilst allowing for inclusion of all participants in the analysis. A pure complete-case analysis would have excluded all patients with any missing values, resulting in substantial loss of information and potential selection bias since missing data cannot be considered at random as well. These constraints limit causal inference but do not undermine the central conclusion that survival differences largely reflect known clinical factors rather than major biological divergence. Finally, this dataset did not include information on each individual hospital to investigate potential regional differences in treatment of eoCRC, aoCRC, and ooCRC patients and the potential impact on survival outcomes. Given the strong centralization of expertise, routine multidisciplinary team meetings, and the national CRC guideline, treatment differences between hospital types are minimized. Notably, treatment differences may also be based on patients’ preferences and shared decision-making.

From a clinical perspective, our results do not support routine escalation of systemic therapy solely on the basis of young age, particularly in patients eligible for standard doublet chemotherapy such as CAPOX or FOLFOX. Instead, treatment decisions should be guided by tumor biology, disease burden, and patient fitness rather than chronological age. This is supported by the reduction in sample size of aoCRC and ooCRC patients in the matched cohort, reflecting that clinical characteristics of eoCRC patients differ from aoCRC and ooCRC patients in real-world clinical practice. In contrast, ooCRC patients clearly represent a vulnerable group with worse outcomes and lower use of combination therapy, underscoring the need for geriatric assessment and age-inclusive clinical trials.

In conclusion, this large nationwide study shows that apparent survival differences in eoCRC are most likely primarily driven by stage at diagnosis and established prognostic factors rather than distinct tumor biology. After accounting for these factors, survival of eoCRC and aoCRC patients is similar. Efforts to reduce mortality from eoCRC should therefore preferably focus on earlier detection, risk stratification, and judicious use of systemic therapy, rather than focusing on age-based treatment escalation.

## Contributors

**Lauri H. Borghuis:** Conceptualization; methodology; software; data curation; formal analysis; investigation; validation; visualization; writing original draft. **Sander R. van Hooff**: Conceptualization; methodology; data curation; formal analysis; resources; writing, review and editing. **Rik van Eekelen:** Methodology; software; data curation; formal analysis; writing, review and editing. **Evelien Dekker:** Writing, review and editing. **Louis Vermeulen:** Conceptualization; methodology; resources; validation; visualization; writing, review and editing; supervision; project administration; funding acquisition. **Tineke E. Buffart:** Conceptualization; methodology; software; data curation; formal analysis; investigation; resources; validation; visualization; writing, review and editing; supervision; project administration; funding acquisition.

Lauri H. Borghuis, Rik van Eekelen, and Tineke E. Buffart had access to the underlying data and were responsible for verifying the accuracy and integrity of the data, analyses, and results reported in the manuscript. All authors read and approved the final version.

## Data sharing statement

The data used in this study were obtained from the NCR, maintained by IKNL. Restrictions apply to the availability of the data, which were used under license for the current study and are not publicly available. Anonymous patient data may be requested upon reasonable request to the corresponding author and according the data access procedures from IKNL. The statistical analysis code is available upon reasonable request to the corresponding author.

## Declaration of interests

**LHB**: Speakers’ fees from Amgen, Pierre Fabre, Servier and Nordic Pharma.

**ED**: Honoraria for consultancy from Olympus, Fujifilm, Norgine, Exact Sciences and ProMedCS. Honoraria for lectures from Olympus, Norgine, IPSEN/Mayoly, FujiFilm, Steris and Pentax. Endoscopic equipment on loan of FujiFilm. Global chair CRC screening committee World Endoscopic Organization (WEO), role within guideline committee for European Society Gastrointestinal Endoscopy (ESGE), European Hereditary Tumor Group (EHTG), and for the National CRC screening program of the Netherlands. All unrelated for this study.

**LV:** Currently employee of Genentech Inc. and shareholder of Roche.

**TEB**: Honoraria for lectures from Amgen, BMS, Pierre Fabre, Servier and Nordic Pharma, advisory board BMS. Research funding from Pierre Fabre, unrelated to this study. Participation on a Data Safety Monitoring Board (DSMB), unrelated to this study.

All other authors declare no competing interests.

## Appendix A Supplementary data

Supplementary Material

Supplemental Captions
