# Supplementary material for: Association between early-onset colorectal cancer outcomes and established prognostic factors: a nationwide population-based study
Source: eClinicalMedicine. 2026 Nov 9;101:104270. doi: 10.1016/j.eclinm.2026.104270 (PMC13770685; doi:10.1016/j.eclinm.2026.104270)
Supplement: Supplementary file 1 [file mmc2.pdf]

**Supplement to:**

Association between early-onset colorectal cancer outcomes and established prognostic factors: a nationwide population-based study

## Table of contents

Scaled Schoenfeld residuals......................................................................................2
Supplementary Figure 1.........................................................................................7
Supplementary Figure 2.........................................................................................8
Supplementary Figure 3.........................................................................................9
Supplementary Figure 4.........................................................................................10
Supplementary Figure 5.........................................................................................11
Supplementary Figure 6.........................................................................................12

Scaled Schoenfeld residual analyses

Given the relatively large sample size of this cohort, the stability of the different covariate effects over time was inspected using scaled Schoenfeld residuals of all the separate covariate effects. Plot of each covariate is depicted below.

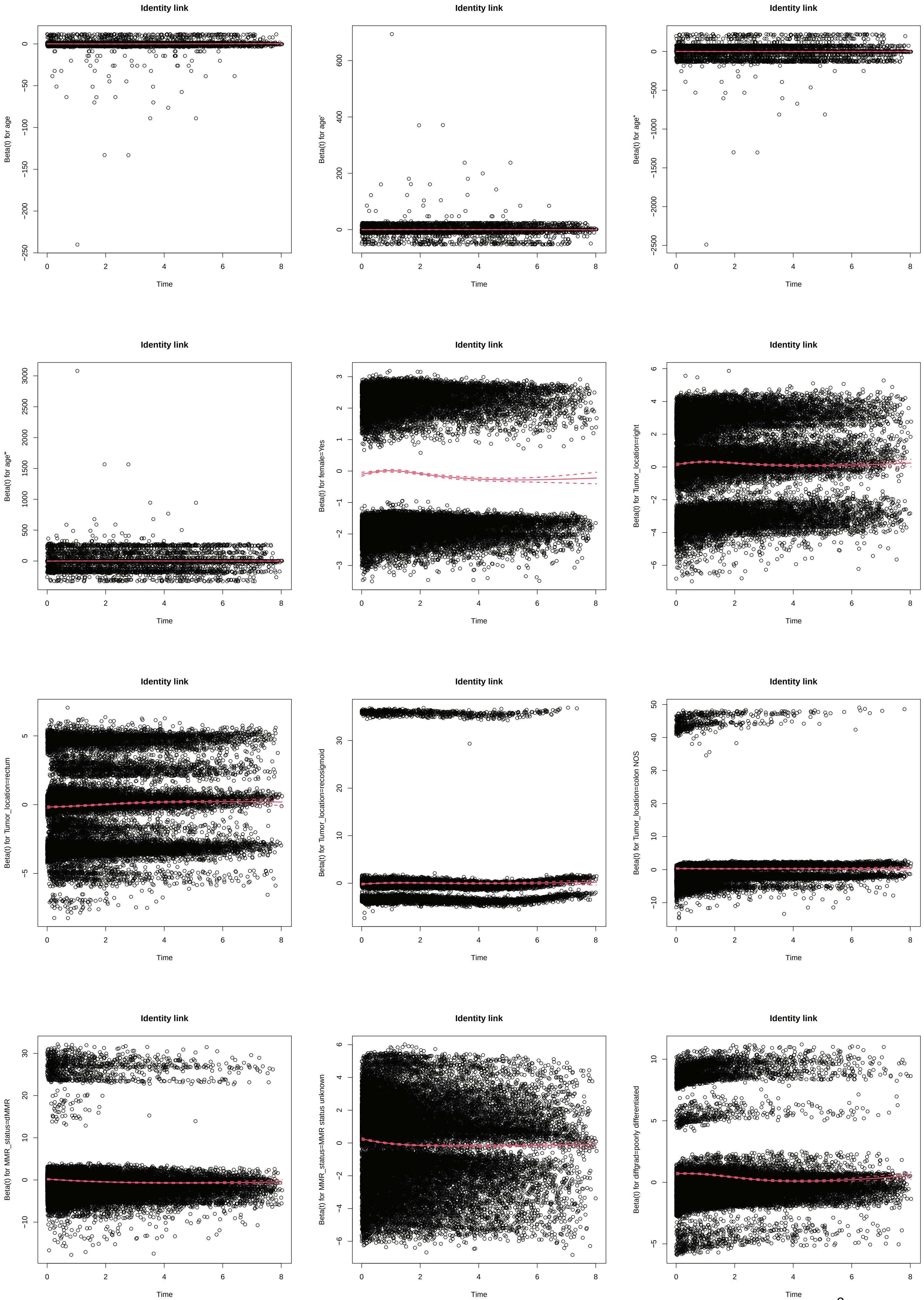
Identity link
Beta(t) for age
Time
Identity link
Beta(t) for age'
Time
Identity link
Beta(t) for age''
Time
Identity link
Beta(t) for age'''
Time
Identity link
Beta(t) for female=Yes
Time
Identity link
Beta(t) for Tumor_location=right
Time
Identity link
Beta(t) for Tumor_location=rectum
Time
Identity link
Beta(t) for Tumor_location=recosigmoid
Time
Identity link
Beta(t) for Tumor_location=colon NOS
Time
Identity link
Beta(t) for MMR_status=dMMR
Time
Identity link
Beta(t) for MMR_status=MMR status unknown
Time
Identity link
Beta(t) for diffgrad=poorly differentiated
Time

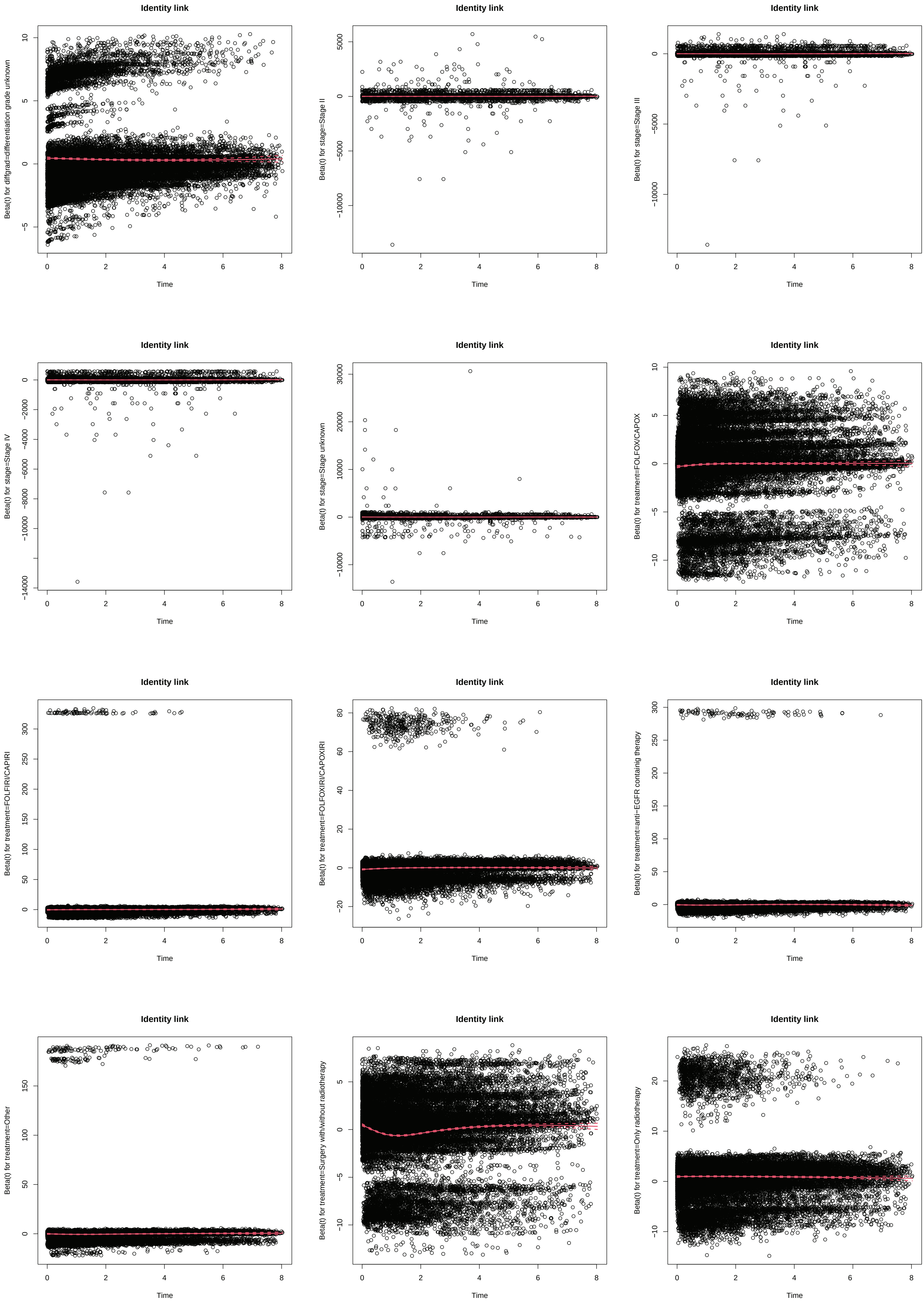
Identity link
Beta(t) for diffgrad=differentiation grade unknown
Time
Identity link
Beta(t) for stage=Stage II
Time
Identity link
Beta(t) for stage=Stage III
Time
Identity link
Beta(t) for stage=Stage IV
Time
Identity link
Beta(t) for stage=Stage unknown
Time
Identity link
Beta(t) for treatment=FOLFOX/CAPOX
Time
Identity link
Beta(t) for treatment=FOLFIRI/CAPIRI
Time
Identity link
Beta(t) for treatment=FOLFOXIRI/CAPOXIRI
Time
Identity link
Beta(t) for treatment=anti−EGFR containing therapy
Time
Identity link
Beta(t) for treatment=Other
Time
Identity link
Beta(t) for treatment=Surgery with/without radiotherapy
Time
Identity link
Beta(t) for treatment=Only radiotherapy
Time

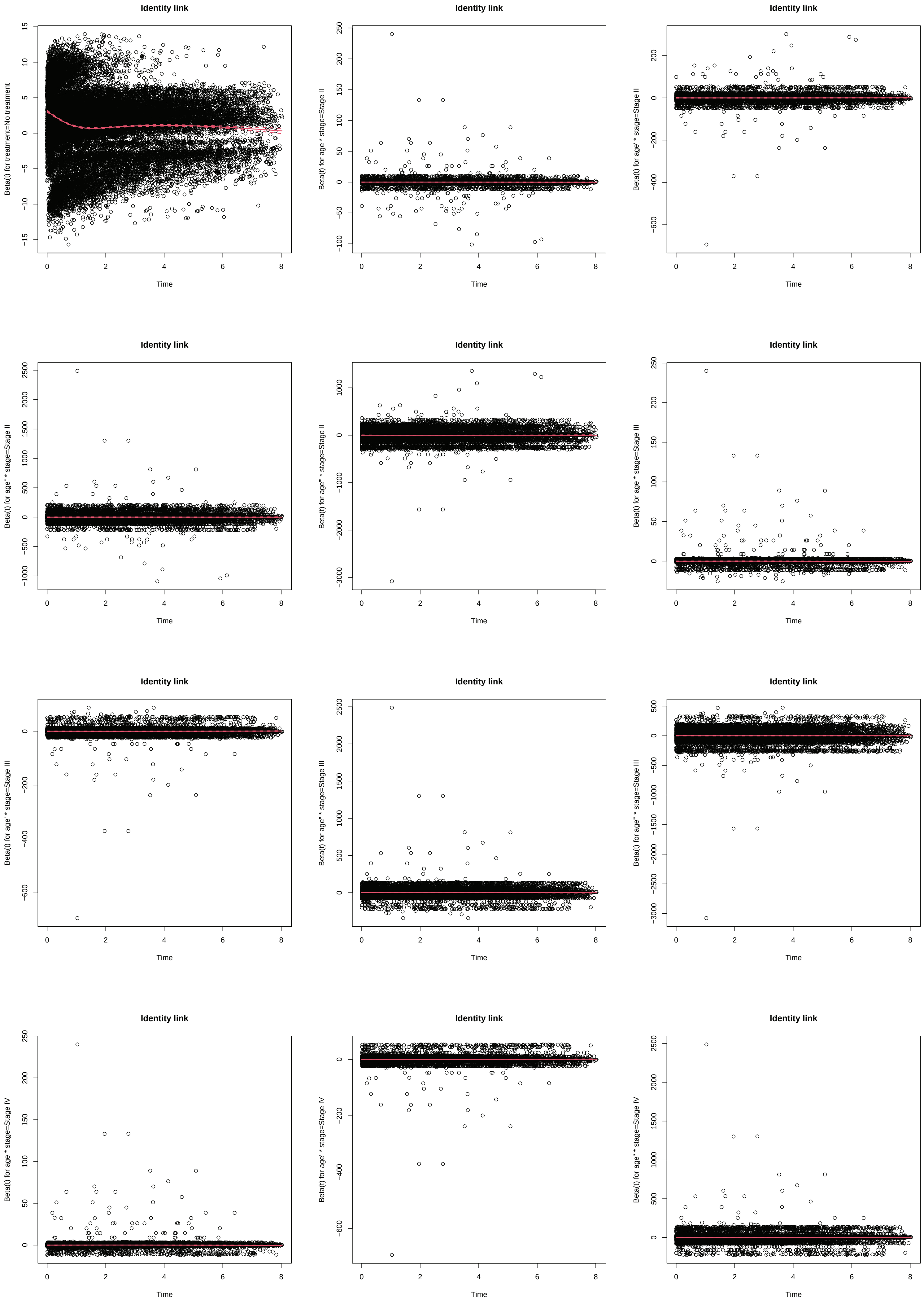
Identity link
Beta(t) for treatment=No treatment
Time
Identity link
Beta(t) for age * stage=Stage II
Time
Identity link
Beta(t) for age' * stage=Stage II
Time
Identity link
Beta(t) for age'' * stage=Stage II
Time
Identity link
Beta(t) for age''' * stage=Stage II
Time
Identity link
Beta(t) for age * stage=Stage III
Time
Identity link
Beta(t) for age' * stage=Stage III
Time
Identity link
Beta(t) for age'' * stage=Stage III
Time
Identity link
Beta(t) for age''' * stage=Stage III
Time
Identity link
Beta(t) for age * stage=Stage IV
Time
Identity link
Beta(t) for age' * stage=Stage IV
Time
Identity link
Beta(t) for age'' * stage=Stage IV
Time

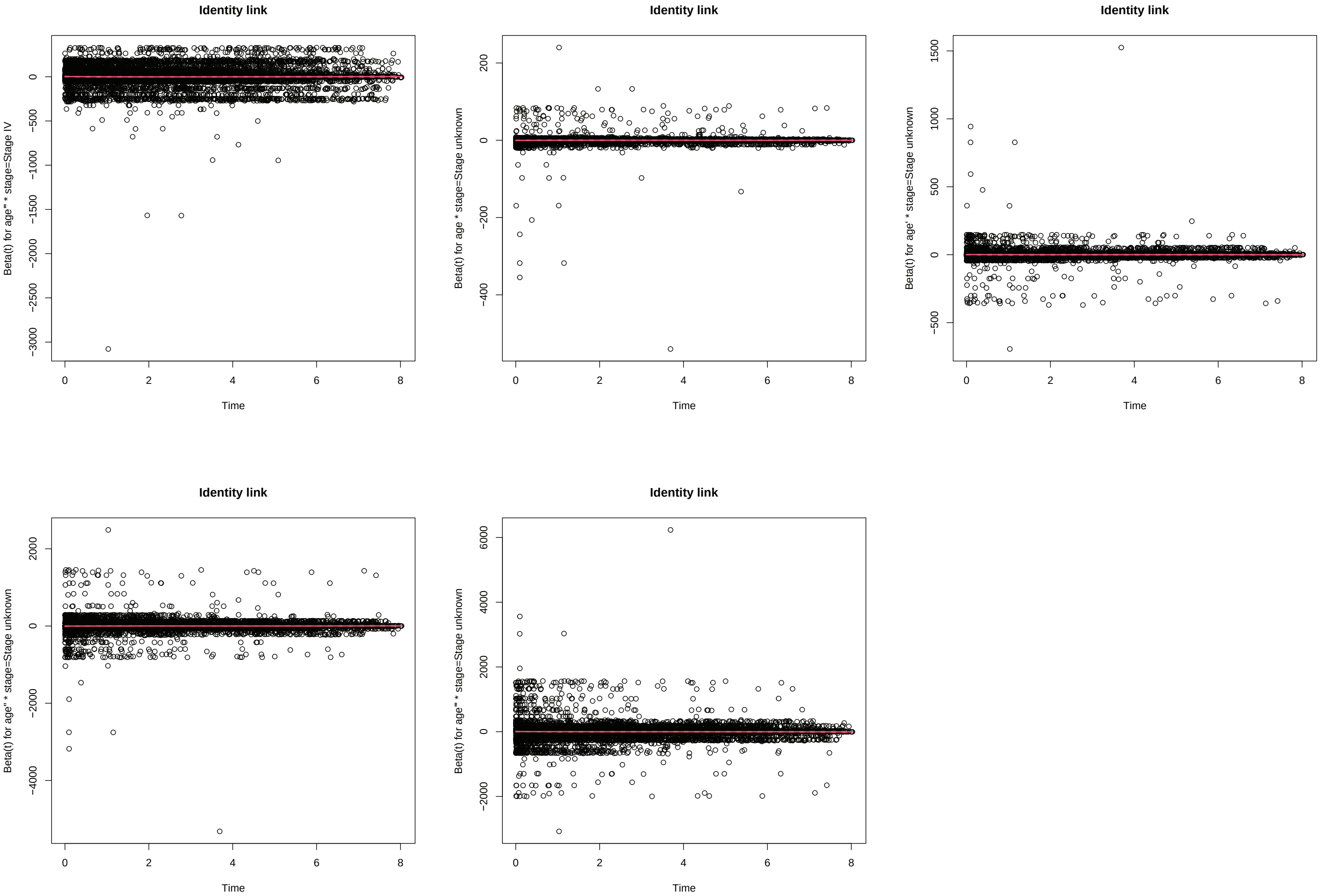

Identity link
Beta(t) for age''' * stage=Stage IV
Time
Identity link
Beta(t) for age * stage=Stage unknown
Time
Identity link
Beta(t) for age' * stage=Stage unknown
Time
Identity link
Beta(t) for age'' * stage=Stage unknown
Time
Identity link
Beta(t) for age''' * stage=Stage unknown
Time

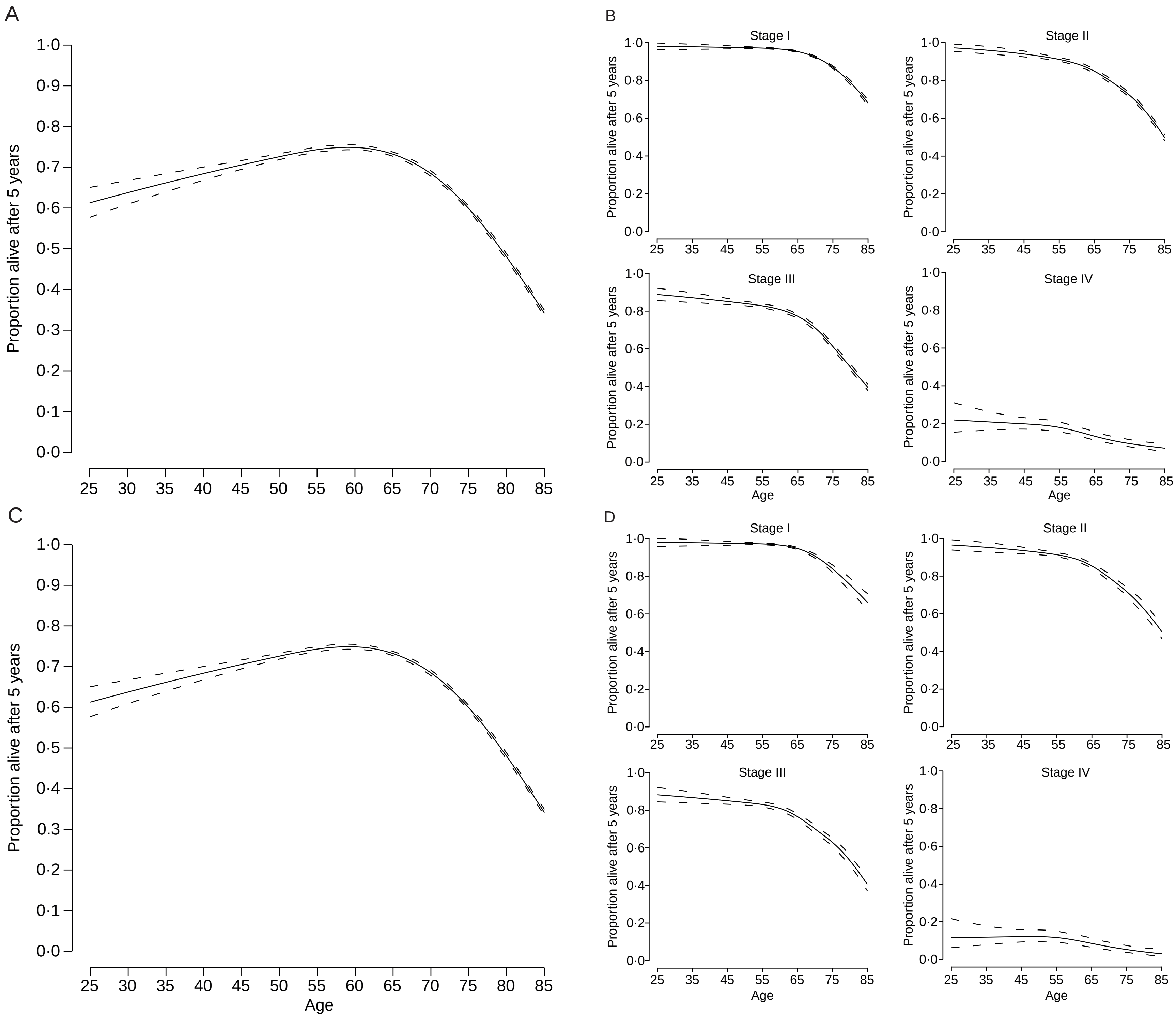


Supplementary Figure 1

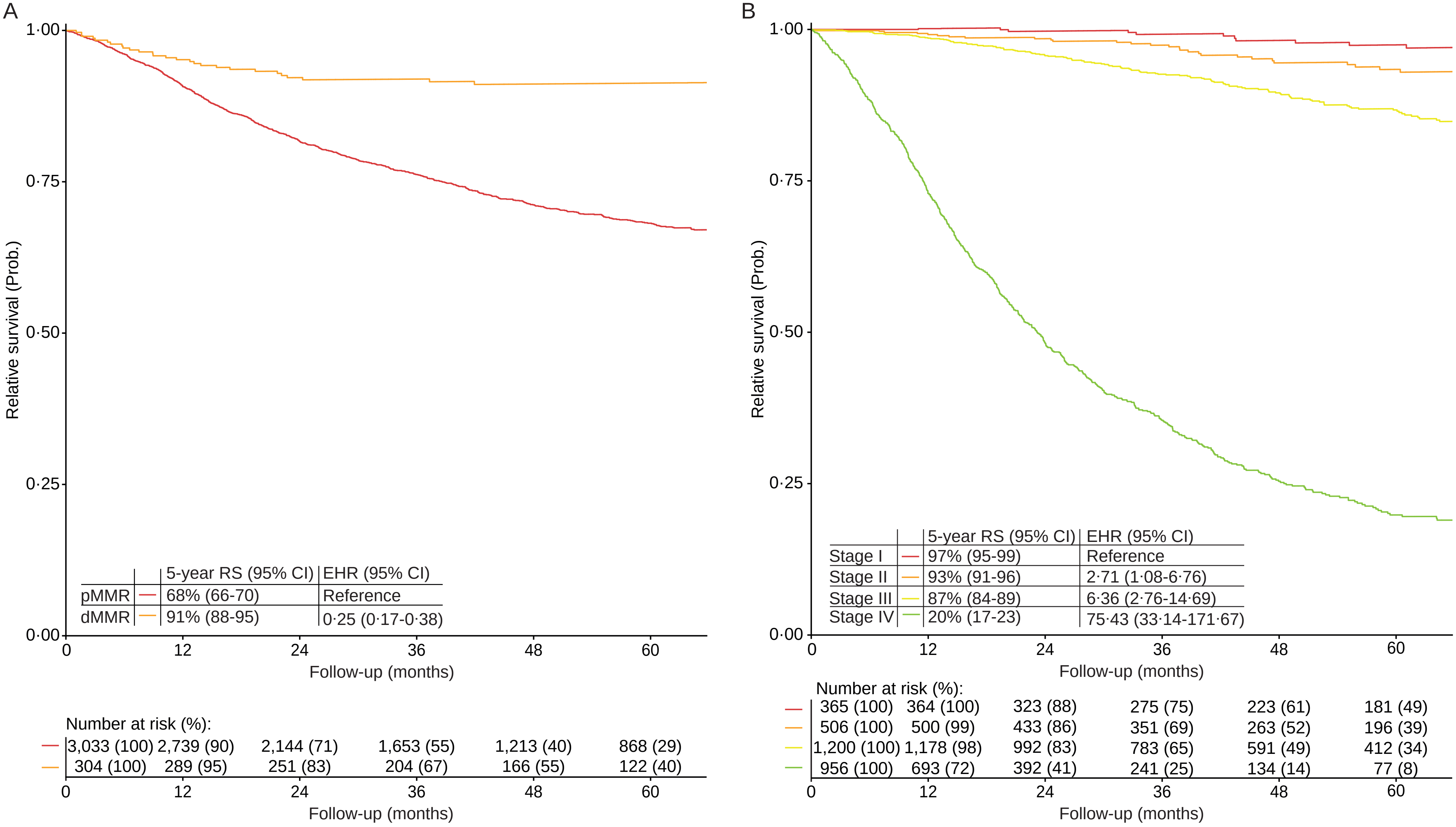


Supplementary Figure 2

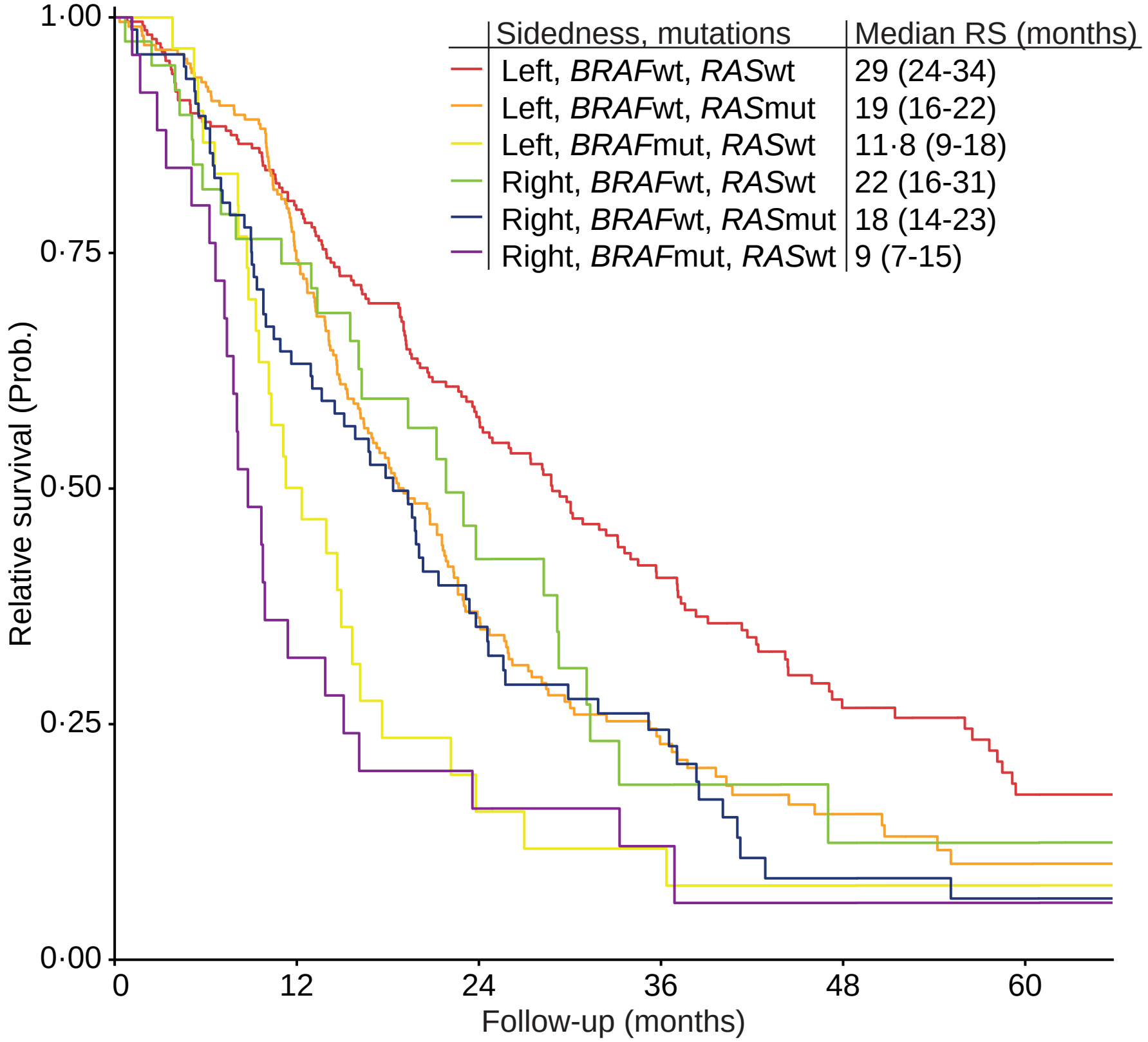


Number at risk n (%):

| Group | 0 | 12 | 24 | 36 | 48 | 60 |
|---|---|---|---|---|---|---|
| Left, *BRAF*wt, *RAS*wt | 215 (100) | 170 (79) | 105 (49) | 60 (28) | 30 (14) | 13 (6) |
| Left, *BRAF*wt, *RAS*mut | 202 (100) | 149 (74) | 58 (29) | 28 (14) | 14 (7) | 7 (3) |
| Left, *BRAF*mut, *RAS*wt | 30 (100) | 15 (50) | 4 (13) | 3 (10) | 2 (7) | 1 (3) |
| Right, *BRAF*wt, *RAS*wt | 39 (100) | 28 (72) | 12 (31) | 4 (10) | 2 (5) | 2 (5) |
| Right, *BRAF*wt, *RAS*mut | 76 (100) | 48 (63) | 24 (32) | 14 (18) | 4 (5) | 2 (3) |
| Right, *BRAF*mut, *RAS*wt | 25 (100) | 8 (32) | 4 (16) | 2 (8) | 1 (4) | 1 (4) |

Follow-up (months)

Supplementary Figure 3

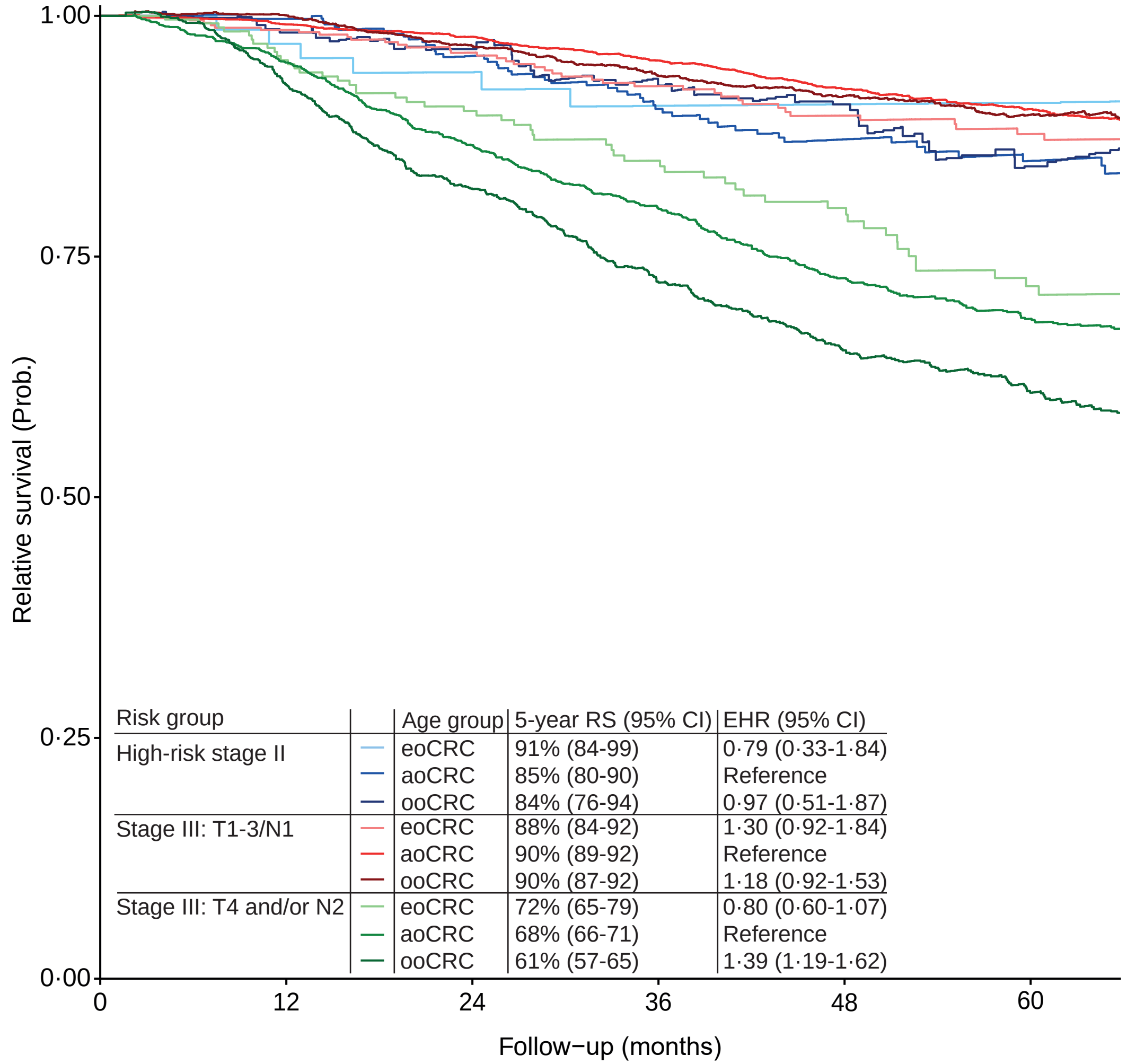


Number at risk n (%):

| | 0 | 12 | 24 | 36 | 48 | 60 |
|---|---|---|---|---|---|---|
| | 66 (100) | 64 (97) | 53 (80) | 44 (68) | 34 (52) | 28 (42) |
| | 328 (100) | 324 (99) | 281 (86) | 220 (67) | 167 (51) | 114 (35) |
| | 183 (100) | 175 (96) | 155 (85) | 117 (64) | 99 (54) | 73 (40) |
| | 377 (100) | 371 (98) | 319 (84) | 256 (68) | 195 (52) | 145 (38) |
| | 3,014 (100) | 2,963 (98) | 2,599 (86) | 2,091 (69) | 1,687 (56) | 1,259 (42) |
| | 1,895 (100) | 1,844 (97) | 1,554 (82) | 1,257 (66) | 1,012 (53) | 745 (39) |
| | 233 (100) | 221 (95) | 185 (79) | 146 (63) | 114 (49) | 81 (35) |
| | 1,525 (100) | 1,440 (94) | 1,151 (75) | 899 (59) | 701 (46) | 523 (34) |
| | 1,067 (100) | 961 (90) | 753 (71) | 557 (52) | 411 (39) | 290 (27) |

Follow−up (months)

Supplementary Figure 4

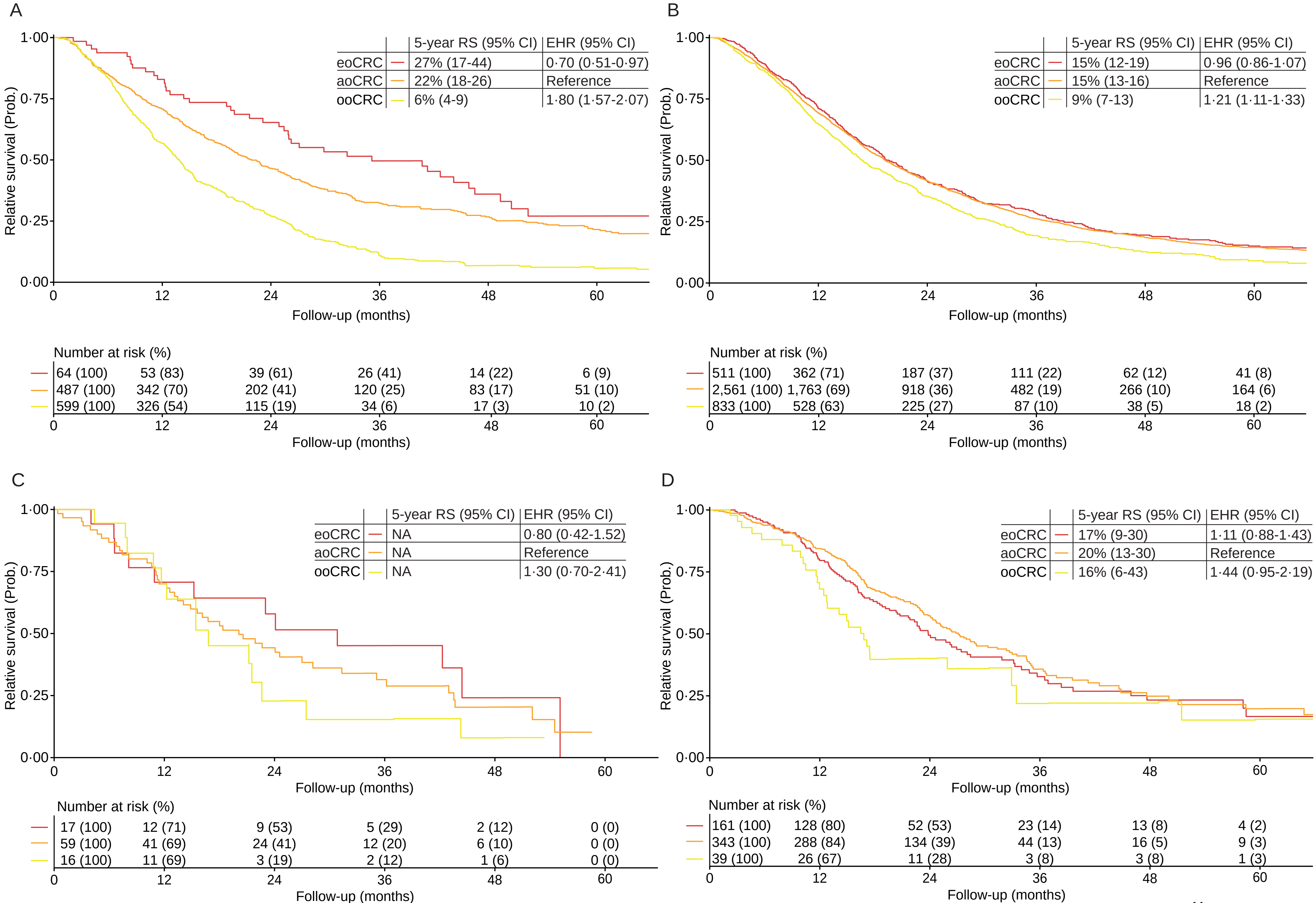


Supplementary Figure 5

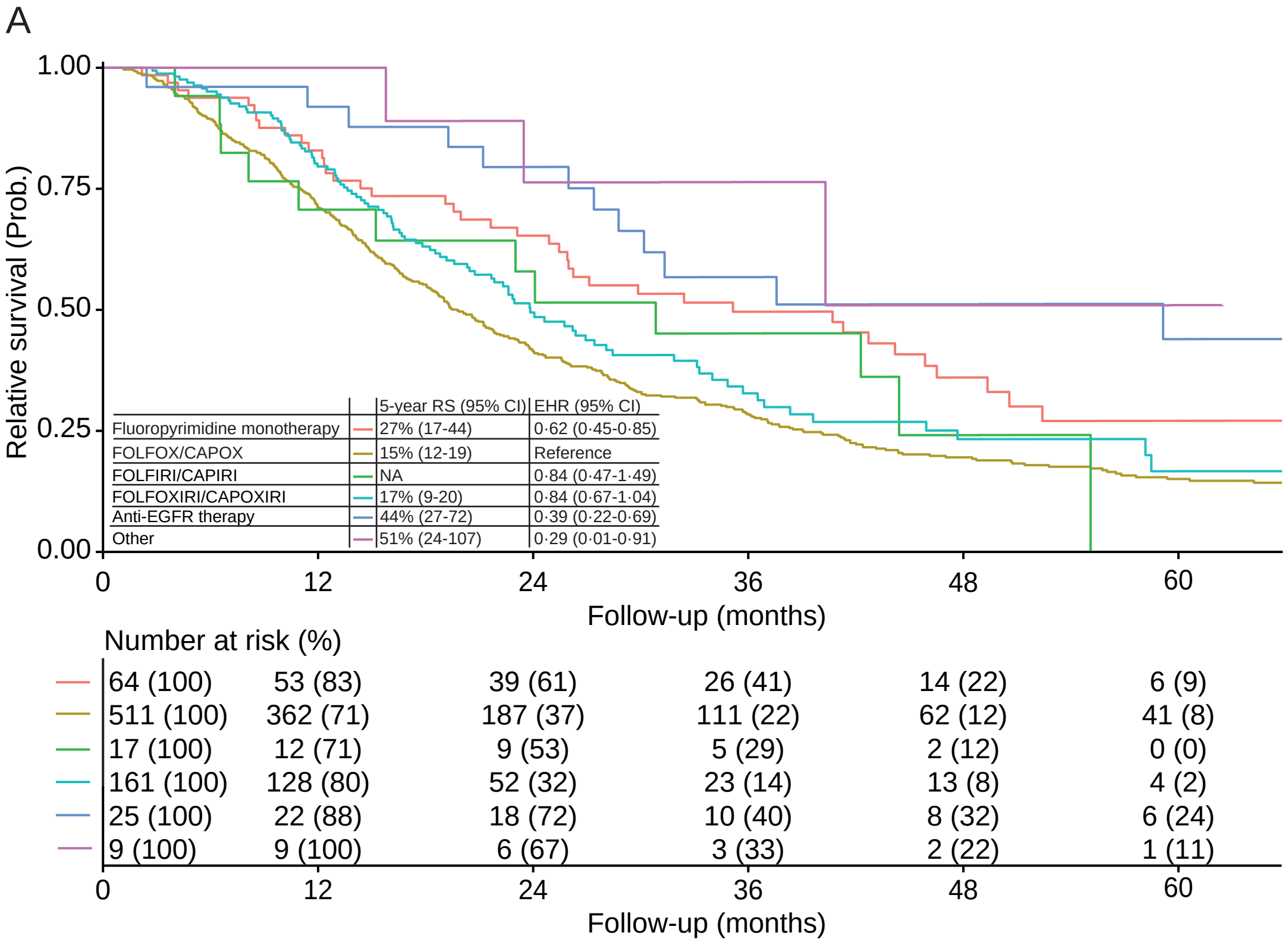

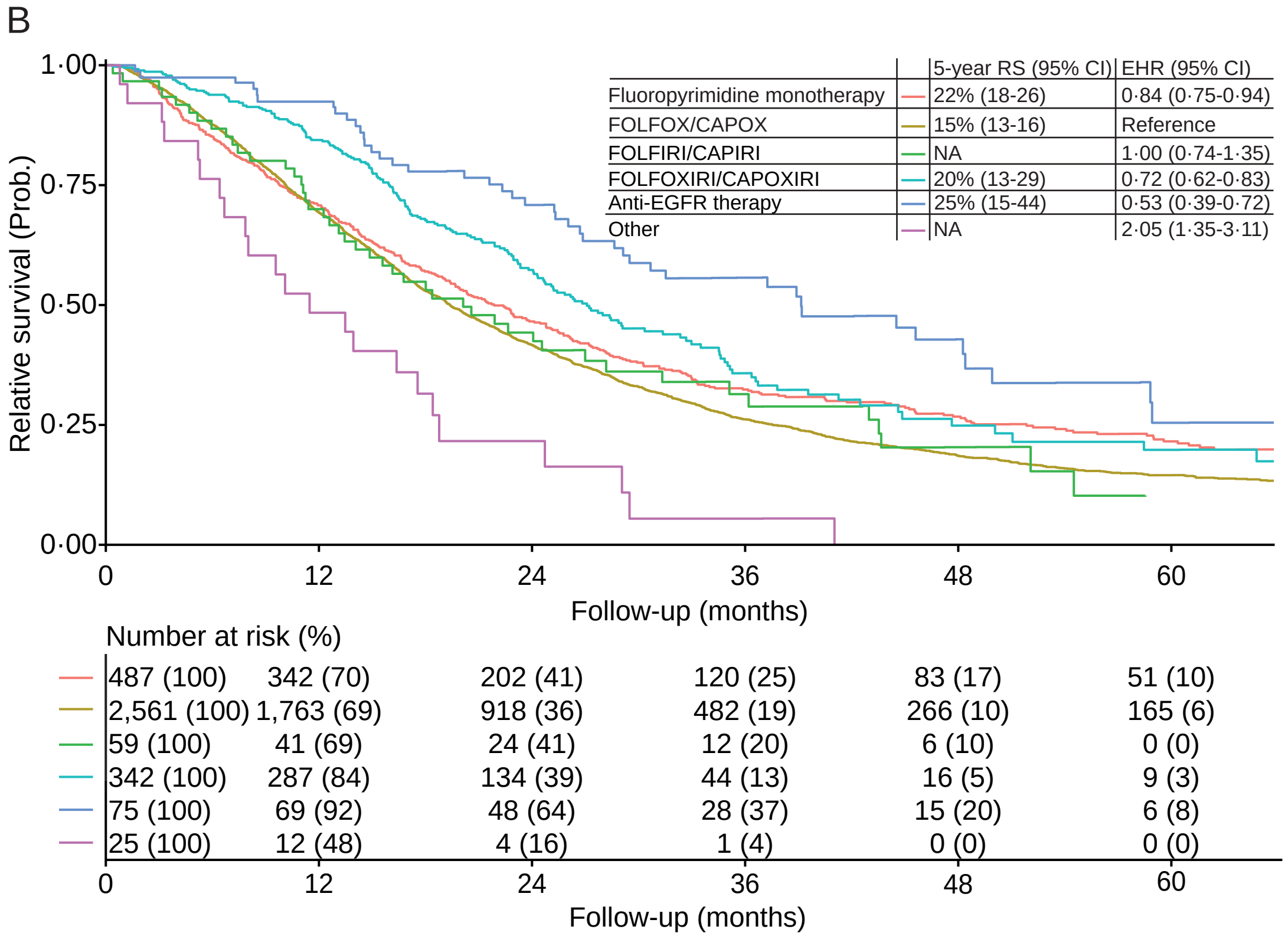

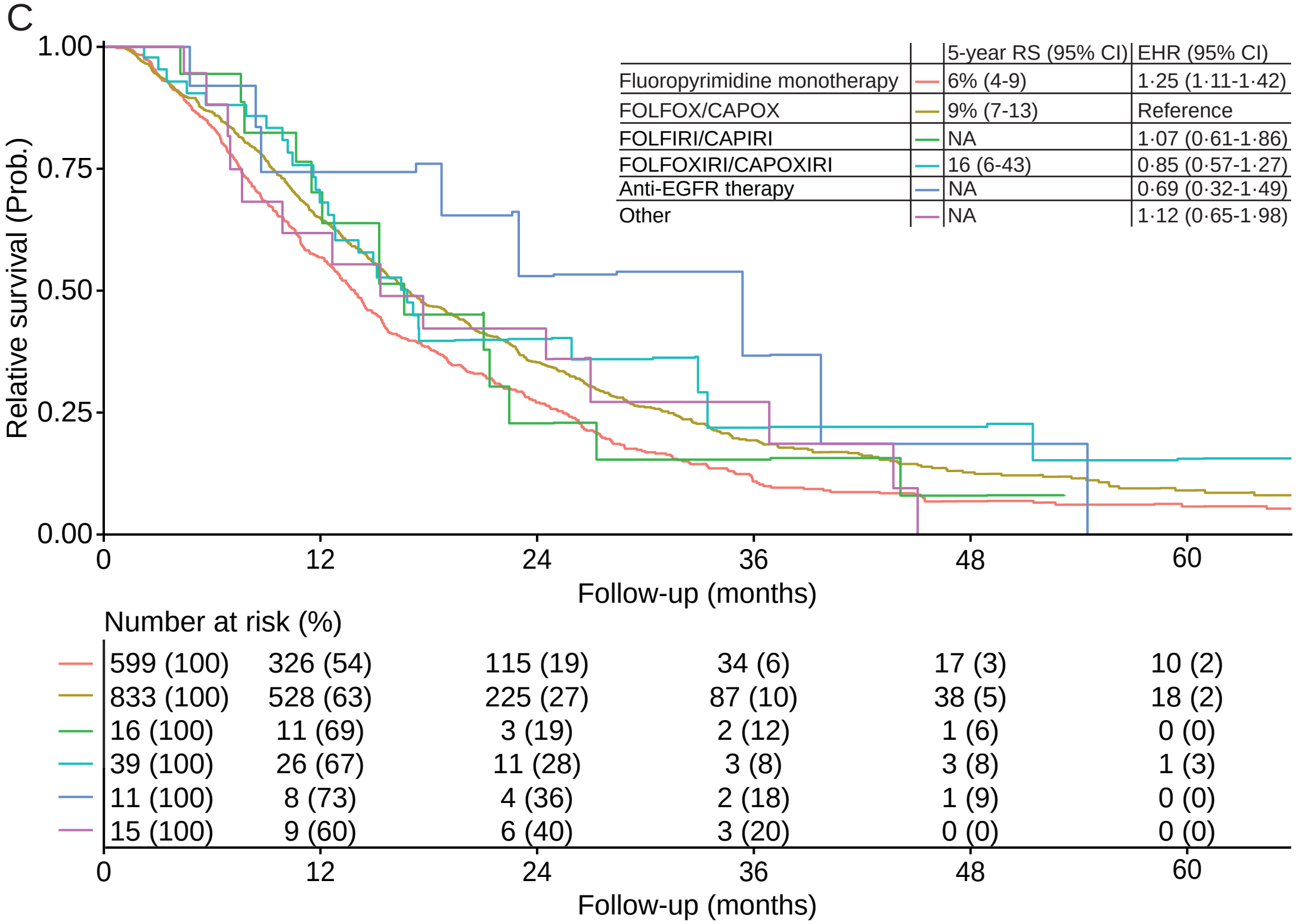


Supplementary Figure 6
