# Supplementary material for: Association between early-onset colorectal cancer outcomes and established prognostic factors: a nationwide population-based study
Source: eClinicalMedicine. 2026 Nov 9;101:104270. doi: 10.1016/j.eclinm.2026.104270 (PMC13770685; doi:10.1016/j.eclinm.2026.104270)
Supplement: Supplementary file 2 [file mmc3.pdf]

Supplementary Figure 1. Cox proportional hazards model for age-specific estimates of 5-year survival

Age-specific estimates of survival at 5 years of follow-up, including 95% CIs, for an individual of age 25 to 85 are plotted. A Cox proportional hazards model with restricted cubic splines for age at diagnosis was fitted as a crude model. For the adjusted model, we also added sex, tumor stage, tumor location, MMR status, grade of differentiation, and treatment as covariates.

A. Crude estimates of 5-year survival for all disease stages (I-IV) combined (after single imputation). **B.** Adjusted estimates of 5-year survival of patients with stage I, II, III or IV at diagnosis (after single imputation). **C.** Crude estimates of 5-year survival for all disease stages (I-IV) combined (complete cases only). **D.** Adjusted estimates of 5-year survival of patients with stage I, II, III or IV at diagnosis (complete cases only).

X-axis: age at diagnosis of CRC; Y axis: proportion of patients alive after 5-years.

Abbreviations: CRC: colorectal cancer CI: confidence interval.

Supplementary Figure 2. Relative survival of eoCRC patients diagnosed with CRC from 1 January 2016 through 31 December 2022 in the Netherlands

A. Kaplan-Meier RS curves of eoCRC patients stratified by MMR status. Crude EHRs for RS were estimated using univariable additive relative survival models with MMR status as the only covariate, using pMMR as the reference category. **B.** Kaplan-Meier RS curves of eoCRC patients with pMMR disease stratified by tumor stage. Crude EHRs for RS were estimated using univariable additive relative survival models with tumor stage as the only covariate, using stage I as the reference category.

Abbreviations: eoCRC: early-onset colorectal cancer; RS: relative survival; EHR: excess hazard ratio; MMR: mismatch repair; dMMR: deficient mismatch repair; pMMR: proficient mismatch repair; CI: confidence interval.

Supplementary Figure 3. Relative survival of eoCRC patients with pMMR stage IV CRC at diagnosis based on tumor sidedness and *RAS/BRAF* mutation status.

Kaplan-Meier RS curves of eoCRC patients with pMMR stage IV CRC stratified by tumor sidedness and *RAS/BRAF* mutation status. Median RS with corresponding 95% CI is shown for each group.

Abbreviations: RS: relative survival; pMMR: proficient mismatch repair; CRC: colorectal cancer; wt: wild type; mut: mutant; CI: confidence interval.

Supplementary Figure 4. Relative survival of the different age groups, based on high-risk stage II, low-risk stage III and high-risk stage III.

Kaplan-Meier RS curves of patients with high-risk stage II, low-risk stage III, and high-risk stage III disease, stratified by three age groups, eoCRC, aoCRC, and ooCRC. Crude EHRs for

relative survival were estimated using univariable additive relative survival models with age groups as the only covariate, with aoCRC patients as the reference category.

Abbreviations: eoCRC: early-onset colorectal cancer; aoCRC: average-onset colorectal cancer; ooCRC: older-onset colorectal cancer; RS: relative survival; EHR: excess hazard ratio; CI: confidence interval.

Supplementary Figure 5. Across-group relative survival of all patients with pMMR stage IV CRC.

A. Kaplan-Meier relative survival (RS) curve of patients with stage IV pMMR CRC treated with fluoropyrimidine monotherapy* (+/- bevacizumab), stratified by eoCRC, aoCRC and ooCRC. Crude excess hazard ratios (EHRs) for RS were estimated using univariable excess mortality models with age groups as the only covariate, aoCRC patients served as the reference category. **B.** Kaplan-Meier RS curve of patients with stage IV pMMR CRC treated with FOLFOX/CAPOX (+/- bevacizumab) stratified by eoCRC, aoCRC and ooCRC. Crude EHRs for RS were estimated using univariable excess mortality models with age groups as the only covariate, again with aoCRC as the reference. **C.** Kaplan-Meier RS curve of patients with stage IV pMMR CRC treated with CAPIRI/FOLFIRI (+/- bevacizumab) stratified by eoCRC, aoCRC and ooCRC. Crude EHRs for RS were estimated using univariable excess mortality models with age groups as the only covariate, again with aoCRC as the reference. **D.** Kaplan-Meier RS curve of patients with stage IV pMMR CRC treated with FOLFOXIRI/CAPOXIRI (+/- bevacizumab) stratified by eoCRC, aoCRC and ooCRC. Crude EHRs for RS were estimated using univariable excess mortality models with age groups as the only covariate, again with aoCRC as the reference.

*Monotherapy: 5-FU/capecitabine/tegafur-gimeracil-oteracil.

Abbreviations: CRC: colorectal cancer; eoCRC: early-onset colorectal cancer; aoCRC: average-onset colorectal cancer; ooCRC: older-onset colorectal cancer; pMMR: proficient mismatch repair; RS: relative survival; EHR: excess hazard ratio; CI: confidence interval; 5-FU: 5-fluorouracil; CAPOX: capecitabine-oxaliplatin; FOLFOX: 5-fluorouracil-leucovorin-oxaliplatin; FOLFIRI: 5-fluorouracil-leucovorin-irinotecan; CAPIRI: capecitabine-irinotecan; FOLFOXIRI: 5-fluorouracil-leucovorin-oxaliplatin-irinotecan; CAPOXIRI; capecitabine-oxaliplatin-irinotecan.

Supplementary Figure 6. Intra-group relative survival of all patients with pMMR stage IV CRC included in the cohort.

A. Kaplan-Meier relative survival (RS) curve of eoCRC patients with stage IV pMMR CRC stratified by treatment fluoropyrimidine monotherapy*, FOLFOX/CAPOX, FOLFIRI/CAPIRI, FOLFOXIRI/CAPOXIRI, all +/- bevacizumab, anti-EGFR therapy** and other. Crude EHRs for RS were estimated using univariable excess mortality models with age groups as the only covariate, CAPOX/FOLFOX served as the reference category. **B.** Kaplan-Meier RS curve of aoCRC patients with stage IV pMMR CRC stratified by treatment fluoropyrimidine monotherapy*, FOLFOX/CAPOX, FOLFIRI/CAPIRI, FOLFOXIRI/CAPOXIRI, all +/- bevacizumab, anti-EGFR therapy** and other. Crude EHRs for relative survival were estimated using univariable excess mortality models with age groups as the only covariate, again with CAPOX/FOLFOX as the reference category. **C.** Kaplan-Meier RS curve of ooCRC patients with pMMR stage IV CRC stratified by treatment fluoropyrimidine monotherapy*, FOLFOX/CAPOX, FOLFIRI/CAPIRI, FOLFOXIRI/CAPOXIRI, all +/- bevacizumab, anti-EGFR therapy** and other. Crude EHRs for relative survival were estimated using univariable excess mortality models with age groups as the only covariate, again with CAPOX/FOLFOX as the reference category

*Monotherapy: 5-FU/capecitabine/tegafur-gimeracil-oteracil.

**Anti-EGFR containing therapy (monotherapy or in combination with chemotherapy).

Abbreviations: CRC: colorectal cancer; eoCRC: early-onset colorectal cancer; aoCRC: average-onset colorectal cancer; ooCRC: older-onset colorectal cancer; pMMR: proficient mismatch repair; RS: relative survival; EHR: excess hazard ratio; CI: confidence interval; 5-FU: 5-fluorouracil; CAPOX: capecitabine-oxaliplatin; FOLFOX: 5-fluorouracil-leucovorin-oxaliplatin; FOLFIRI: 5-fluorouracil-leucovorin-irinotecan; CAPIRI: capecitabine-irinotecan; FOLFOXIRI: 5-fluorouracil-leucovorin-oxaliplatin-irinotecan; CAPOXIRI; capecitabine-oxaliplatin-irinotecan; EGFR: Epidermal growth factor receptor.
