# Supplementary material for: Association between early-onset colorectal cancer outcomes and established prognostic factors: a nationwide population-based study
Source: eClinicalMedicine. 2026 Nov 9;101:104270. doi: 10.1016/j.eclinm.2026.104270 (PMC13770685; doi:10.1016/j.eclinm.2026.104270)
Supplement: Supplementary file 3 [file mmc1.docx]

| Supplementary Table 1: Clinicopathological characteristics of all eoCRC patients diagnosed with pMMR and dMMR adenocarcinoma from 1 January 2016 through 31 December 2022. | | | |  |
| --- | --- | --- | --- | --- |
|  | pMMR (n= 3,033)  n (%) | dMMR (n= 304)  n (%) | SMD* | |
| **Age (years)**  Median (IQR) | 45 (41-48) | 42 (37-47) | 0·46 | |
| **Sex**  Male  Female | 1,513 (50)  1,520 (50) | 182 (60)  122 (40) | 0·20 | |
| **Tumor location**  Right colon  Left colon  Rectosigmoid  Rectum  Colon NOS | 557 (18)  1,170 (39)  103 (3)  1,177 (39)  26 (1) | 174 (57)  75 (25)  5 (2)  46 (15)  4 (1) | 0·91 | |
| **Stage****  Stage I  Stage II  Stage III  Stage IV  Unknown | 365 (12)  506 (17)  1,200 (40)  956 (31)  6 | 40 (13)  100 (33)  121 (40)  42 (14)  1 | 0·51^a^ | |
| **Surgical resection (stage I-III only)**  Yes  No | **n = 2,071**  1,876 (91)  195 (9) | **n = 261**  242 (93)  19 (7) | 0·08 | |
| **Treatment (stage I-III only)**  Surgery alone  (Neo)adjuvant chemotherapy + CRT (+/- surgery)  (Neo)adjuvant chemotherapy + RT (+/- surgery)  (Neo)adjuvant CRT +/- surgery  (Neo)adjuvant RT +/- surgery  (Neo)adjuvant chemotherapy + surgery  Systemic therapy without surgery  Other  No treatment  Unknown | **n = 2,089**  711 (34)  345 (17)  28 (1)  99 (5)  121 (6)  677 (33)  21 (1)  7 ()  2 (0)  60 (3) | **n = 261**  124 (48)  24 (9)  0 ()  9 (3)  4 (2)  91 (35)  7 (3)  0 ()  0 ()  2 (1) | 0·48 | |
| **Local treatment (stage IV only)**  Surgery/ablation metastases  Radiotherapy metastases  Surgery/ablation + radiotherapy metastases  No local treatment | **n = 956**  324 (34)  33 (3)  13 (1)  586 (61) | **n = 42**  15 (36)  0 ()  0 ()  27 (64) | 0·32 | |
| **Systemic treatment (stage IV only)^b^**  Fluoropyrimidine monotherapy^c^  FOLFOX/CAPOX  FOLFIRI/CAPIRI  FOLFOXIRI/CAPOXIRI  Anti-EGFR therapy^d^  Other  No systemic therapy | **n = 956**  64 (7)  511 (53)  17 (2)  161 (17)  25 (3)  9 (1)  169 (18) | **n = 42**  0 ()  12 (29)  1 (2)  4 (10)  0 ()  13 (31)  12 (28) | 1·15 | |

Data are n (%). * SMD values closer to 0 indicate smaller differences between age groups. ** Data not available for all patients. a = SMD was assessed excluding cases with unknown values. b = all systemic treatments are +/- bevacizumab, except for anti-*EGFR* therapy. c = 5-FU/capecitabine/tegafur-gimeracil-oteracil. d = Anti-*EGFR* containing therapy (monotherapy or in combination with chemotherapy). Abbreviations: eoCRC: early-onset colorectal cancer; dMMR: deficient mismatch repair; pMMR: proficient mismatch repair; SMD: Standard mean difference; IQR: interquartile range; NOS: not otherwise specified; CRT: chemoradiotherapy; RT: radiotherapy; 5-FU: 5-fluorouracil; FOLFOX: 5-fluorouracil-leucovorin-oxaliplatin; CAPOX: capecitabine-oxaliplatin; FOLFIRI: 5-fluorouracil-leucovorin-irinotecan; CAPIRI: capecitabine-irinotecan; FOLFOXIRI: 5-fluorouracil-leucovorin-oxaliplatin-irinotecan; CAPOXIRI; capecitabine-oxaliplatin-irinotecan; *EGFR:* epidermal growth factor receptor.

| Supplementary Table 2: Clinicopathological characteristics of patients with high-risk stage II, low-risk stage III or high-risk stage III disease. | | | | | | | | | | | | | | | | |
| --- | --- | --- | --- | --- | --- | --- | --- | --- | --- | --- | --- | --- | --- | --- | --- | --- |
|  | **High-risk stage II (T4/N0)** | | | |  | **Low-risk stage III (T1-3/N1)** | | | | | **High-risk stage III (T4 and/or N2)** | | | |  |  |
|  | eoCRC  (n=93)  n (%) | aoCRC (n=638)  n (%) | ooCRC  (n= 1,279)  n (%) | SMD* <50 vs 50-69 | SMD* 50-69 vs ≥70 | eoCRC (n=519)  n (%) | aoCRC (n=4,263)  n (%) | ooCRC  (n= 4,889)  n (%) | SMD* <50 vs 50-69 | SMD* 50-69 vs  ≥70 | eoCRC  (n=251)  n (%) | aoCRC (n=1,770)  n (%) | ooCRC  (n= 2,137)  n (%) | SMD* <50 vs 50-69 | SMD* 50-69 vs ≥70 |  |
| Age (years)  Median (IQR) | 44 (40-47) | 63 (57-66) | 79 (75-83) |  |  | 45 (41-48) | 62 (57-66) | 76 (73-81) |  |  | 45 (40-48) | 61 (56-66) | 77 (73-82) |  |  |  |
| Sex  Male  Female | 38 (41)  55 (59) | 313 (49)  325 (51) | 607 (47)  672 (53) | 0·17 | 0·03 | 232 (45)  287 (55) | 2,341 (55)  1,922 (45) | 2,666 (55)  2,223 (45) | 0·21 | 0·01 | 123 (49)  128 (51) | 976 (55)  794 (45) | 1,108 (52)  1,029 (48) | 0·12 | 0·07 |  |
| Tumor location  Right colon  Left colon  Rectosigmoid^a^  Colon NOS | 36 (39)  56 (60)  0 (0)  1 (1) | 347 (54)  274 (43)  11 (2)  6 (1) | 759 (60)  493 (39)  18 (1)  9 (1) | 0·20 | 0·09 | 158 (31)  333 (64)  22 (4)  6 (1) | 1,649 (39)  2,413 (57)  166 (4)  35 (1) | 2,601 (53)  2,047 (42)  170 (4)  71 (1) | 0·13 | 0·28 | 83 (33)  153 (61)  13 (5)  2 (1) | 769 (43)  919 (52)  69 (4)  13 (1) | 1,315 (62)  751 (35)  47 (2)  24 (1) | 0·23 | 0·36 |  |
| MMR status**  dMMR  pMMR  Unknown | 13 (16)  69 (84)  11 | 92 (16)  476 (84)  70 | 162 (29)  404 (71)  713 | 0·01^b^ | 0·30^b^ | 46 (10)  422 (90)  51 | 298 (8)  3,447 (92)  518 | 349 (15)  2,048 (85)  2,492 | 0·07^b^ | 0·21^b^ | 14 (6)  216 (94)  21 | 141 (9)  1,422 (91)  207 | 213 (20)  877 (80)  1,047 | 0·11^b^ | 0·30^b^ |  |
| (Neo)adjuvant therapy  Yes  5FU/capecitabine  FOLFOX/CAPOX^c^  Other^d^  No | 67 (72)  4 (1)  62 (98)  1 (1)  26 (28) | 330 (52)  14 (4)  313 (95)  3 (1)  308 (48) | 186 (15)  69 (37)  113 (61)  4 (2)  1,093 (85) | 0·44 | 0·99 | 460 (89)  9 (2)  411 (89)  40 (9)  59 (11) | 3,455 (81)  248 (7)  3,106 (90)  101 (3)  808 (19) | 2,324 (47)  940 (41)  1,348 (57)  36 (2)  2,565 (53) | 0·39 | 1·07 | 234 (93)  6 (3)  219 (93)  9 (4)  17 (7) | 1,527 (86)  82 (5)  1,424 (93)  21 (2)  243 (14) | 1,070 (50)  412 (39)  645 (60)  13 (1)  1,067 (50) | 0·31 | 1·19 |  |

Data are n (%). * SMD values closer to 0 indicate smaller differences between age groups. ** Data not available for all patients. a = Patients that did not receive radiotherapy/chemoradiotherapy. b = SMD was assessed excluding cases with unknown values. c = FOLFOX/CAPOX +/- bevacizumab. d = Other treatments: FOLFIRI/CAPIRI/FOLFOXIR/CAPOXIRI +/- bevacizumab. Abbreviations: SMD: standard mean difference; IQR: interquartile range; MMR: Mismatch repair; dMMR: deficient mismatch repair; pMMR: proficient mismatch repair; 5FU: 5-fluorouracil; FOLFOX: 5-fluorouracil-leucovorin-oxaliplatin; CAPOX: capecitabine-oxaliplatin; FOLFIRI: 5-fluorouracil-leucovorin-irinotecan; CAPIRI: capecitabine-irinotecan; FOLFOXIRI: 5-fluorouracil-leucovorin-oxaliplatin-irinotecan; CAPOXIRI; capecitabine-oxaliplatin-irinotecan.

| Supplementary Table 3: Clinical characteristics of all patients in the matched cohort. | | | | | |
| --- | --- | --- | --- | --- | --- |
|  | eoCRC (n=2,830)  n (%) | aoCRC (n=2,604)  n (%) | ooCRC (n=1,995)  n (%) | SMD*  <50 vs 50-69 | SMD*  50-69 vs ≥70 |
| Age (years)  Median (IQR) | 45 (41-48) | 61 (56-65) | 74 (71-77) |  |  |
| Sex  Male  Female | 1,419 (50)  1,411 (50) | 1,506 (58)  1,098 (42) | 1,263 (63)  732 (37) | 0·16 | 0·11 |
| Tumor location  Right colon  Left colon  Rectosigmoid  Rectum  Colon NOS | 513 (18)  1,092 (38)  103 (4)  1,098 (39)  24 (1) | 475 (18)  1,021 (39)  92 (4)  996 (38)  20 (1) | 425 (21)  798 (40)  62 (3)  694 (35)  16 (1) | 0·02 | 0·09 |
| Grade of differentiation**  Well/moderately  Poor/undifferentiated  Unknown | 2,366 (91)  245 (9)  219 | 2,208 (91)  207 (9)  189 | 1,695 (92)  154 (8)  146 | 0·03^a^ | 0·01^a^ |
| Stage**  Stage I  Stage II  Stage III  Stage IV  Unknown | 342 (12)  470 (17)  1,119 (40)  893 (31)  6 | 332 (13)  450 (17)  1034 (40)  784 (30)  4 | 293 (15)  396 (20)  742 (37)  562 (28)  2 | 0·04^a^ | 0·10^a^ |

Data are n (%). * SMD values closer to 0 indicate smaller differences between age groups. ** Data not available for all patients. a = SMD was assessed excluding cases with unknown values. Abbreviations: SMD: Standard mean difference; IQR: interquartile range; NOS: not otherwise specified

**Supplementary Table 4:** Characteristics of patients with stage IV disease at diagnosis in the matched cohort.

|  | eoCRC (n=893)  n (%) | aoCRC (n=784)  n (%) | ooCRC (n=562)  n (%) | SMD*  <50 vs 50-69 | SMD*  50-69 vs ≥70 |
| --- | --- | --- | --- | --- | --- |
| Age (years)  Median (IQR) | 45 (41-48) | 59 (55-64) | 74 (72-78) |  |  |
| Sex  Male  Female | 461 (52)  432 (48) | 459 (58)  325 (42) | 370 (66)  192 (34) | 0·14 | 0·15 |
| Tumor location  Right colon  Left colon  Rectosigmoid  Rectum  Colon NOS | 195 (22)  348 (39)  32 (4)  308 (34)  10 (1) | 180 (23)  311 (40)  23 (3)  261 (33)  9 (1) | 152 (27)  223 (40)  14 (3)  166 (29)  7 (1) | 0·05 | 0.11 |
| Grade of differentiation**  Well/moderately  Poor/undifferentiated  Undefined | 621 (82)  139 (18)  133 | 551 (84)  107 (16)  126 | 405 (86)  67 (14)  90 | 0·05^a^ | 0.06^a^ |
| *RAS***  Wild type  Mutation  Unknown*** | 297 (48)  319 (52)  277 | 249 (48)  272 (52)  263 | 174 (48)  192 (52)  196 | 0·01^a^ | 0·01^a^ |
| *BRAF***  Wild type  Mutation  Unknown*** | 493 (89)  61 (11)  339 | 423 (90)  47 (10)  314 | 297 (90)  32 (10)  232 | 0·03^a^ | 0·01^a^ |
| Local treatment  Surgery/ablation metastases  Radiotherapy metastases  Surgery + radiotherapy metastases  No local treatment | 303 (34)  32 (4)  12 (1)  546 (61) | 269 (34)  21 (3)  9 (1)  485 (62) | 170 (3)  9 (2)  6 (1)  377 (67) | 0·06 | 0·12 |
| Systemic treatment^b^  Fluoropyrimidine monotherapy^c^  FOLFOX/CAPOX  FOLFIRI/CAPIRI  FOLFOXIRI/CAPOXIRI  Anti-*EGFR* therapy^d^  Other  No systemic therapy | 62 (7)  486 (54)  16 (2)  136 (15)  23 (3)  9 (1)  161 (18) | 57 (7)  473 (60)  4 (1)  82 (11)  12 (2)  1 (0)  155 (20) | 74 (13)  323 (58)  4 (1)  23 (4)  5 (1)  1 (0)  132 (23) | 0·24 | 0·32 |

Data are n (%). * SMD values closer to 0 indicate smaller differences between age groups. ** Data not available for all patients. a = SMD was assessed excluding cases with unknown values. ***For RAS, 31%, 34%, and 41% of eoCRC, aoCRC, and ooCRC was missing. For BRAF, 38%, 40%, and 35% of eoCRC, aoCRC, and ooCRC was missing. b = all systemic treatments are +/- bevacizumab, except for anti-*EGFR* therapy. c = 5-FU/capecitabine/tegafur-gimeracil-oteracil. d = Anti-*EGFR* containing therapy (monotherapy or in combination with chemotherapy). Abbreviations: CRC: colorectal cancer; eoCRC: early-onset colorectal cancer; aoCRC: average-onset colorectal cancer; ooCRC: older-onset colorectal cancer; pMMR: proficient mismatch repair; IQR: interquartile range; NOS: not otherwise specified; 5-FU: 5-fluorouracil; FOLFOX: 5-fluorouracil-leucovorin-oxaliplatin; CAPOX: capecitabine-oxaliplatin; FOLFIRI: 5-fluorouracil-leucovorin-irinotecan; CAPIRI: capecitabine-irinotecan; FOLFOXIRI: 5-fluorouracil-leucovorin-oxaliplatin-irinotecan; CAPOXIRI; capecitabine-oxaliplatin-irinotecan; *EGFR*: epidermal growth factor receptor.
